# High Intratumoral PROS1 Expression Is Associated with Improved Survival, Reduced Hypoxia Score Markers, and Alterations in Gene Expression Associated with Oncogene Signaling and Cell Cycle Signaling in Selected Solid Tumors

**DOI:** 10.3390/ijms27219613

**Published:** 2026-10-27

**Authors:** Teagan Prouse, Rinku Majumder, Samarpan Majumder

**Affiliations:** 1 Department of Interdisciplinary Oncology, Louisiana State University Health Sciences Center, New Orleans, LA 70112, USA; tprous@lsuhsc.edu (T.P.); rmajum@lsuhsc.edu (R.M.); 2 Department of Microbiology, Genetics & Immunology, Louisiana State University Health Sciences Center, New Orleans, LA 70112, USA

**Keywords:** Protein S, *PROS1*, pancreatic adenocarcinoma, NSCLC adenocarcinoma, ovarian serous cystadenocarcinoma

## Abstract

Protein S (PS), encoded by *PROS1*, is a vitamin K-dependent physiological anticoagulant with emerging, context-dependent roles in tumor biology. In pancreatic ductal adenocarcinoma (PDAC), exogenous PS suppresses tumor growth and intratumoral hypoxia in preclinical models. Notably, high intratumoral *PROS1* expression is associated with improved survival and attenuation of oncogenic, hypoxia-responsive, and EMT-related transcriptional programs in PDAC. Here, we examined associations between intratumoral *PROS1* expression and overall survival, hypoxia-associated signaling, and genomic instability across nine cancer types using publicly available cBioPortal datasets. Tumors were stratified by low versus high *PROS1* expression. Genomic instability was assessed using tumor mutational burden, aneuploidy, and tumor break load, where available. High *PROS1* expression was associated with improved survival in ovarian serous cystadenocarcinoma and lung adenocarcinoma in exploratory, unadjusted Mantel-Haenszel hazard ratio analyses. It was also inversely associated with genomic instability markers in ovarian, lung adenocarcinoma, and PDAC. Meta-analyses in select genes under low and high *PROS1* expression conditions revealed shifts in pathways linked to oncogenic signaling, cell-cycle regulation, hypoxia, and EMT. Collectively, these findings position *PROS1* as a candidate prognostic biomarker in selected cancers and support further investigation of *PROS1*’s roles in limiting tumor progression and preserving genomic stability.

## 1. Introduction

Protein S (PS), encoded by *PROS1*, is a 75 KDa vitamin K-dependent plasma glycoprotein best known for its role in negative regulation of the coagulation cascade. It is found naturally in two physiologically active forms: free or bound to complement-4 binding protein 4 (C4BP), an acute phase protein [1]. The physiological concentration of PS is 350 nM, 60% of which is bound to C4BP [2] and the other 40% is free in plasma [3]. Free PS is considered to have the closest association with thrombotic risk [4]. Protein S is a γ-carboxyglutamate-containing plasma glycoprotein created and secreted primarily by hepatocytes [5], with additional production by endothelial cells [6], Leydig cells [7], and, at low levels, by T cells, where it participates in signaling [8,9].

In the coagulation cascade, PS functions as a cofactor for activated Protein C and amplifies the inactivation of factor Va and factor VIIIa (FVIIIa) [10,11,12]. Protein S is also a cofactor of Tissue Factor Pathway Inhibitor (TFPI) and inhibits factor Xa with TFPI by 4- to 10-fold [13,14,15]. Independently, PS directly inhibits factor IXa (FIXa) activity both in the presence and absence of FVIIIa [16,17] and regulates the propagation phase of coagulation, independent of APC [18]. These anticoagulant properties are particularly relevant to cancer, in which hypercoagulability, intratumoral thrombosis, vascular dysfunction, hypoperfusion, and hypoxia can reinforce tumor progression and therapeutic resistance [1,19,20,21,22].

Beyond hemostasis, PS regulates cellular processes relevant to cancer biology, including proliferation, migration, phagocytosis, vascular hemostasis, and immune signaling [1,21]. These functions are mediated in part through TAM (TYRO3, AXL, and MERTK) family receptor tyrosine kinases [21]. Consequently, *PROS1* expression has been associated with either tumor-promoting or tumor-suppressive activity depending on the tumor type, cellular context, receptor repertoire, and microenvironmental state. This context dependent associations between *PROS1* expression and tumor behavior have been reported in breast carcinoma [23], lung cancer [24], glioblastoma [25], prostate carcinoma [26], hepatocellular carcinoma [27], thyroid cancer [23], endometrial carcinoma [28], uveal melanoma [29], sarcoma [30], and squamous head and neck cancer [31].

Notably, PS has been reported to act as a potent anti-tumor agent, and intratumoral expression influences patient survival in PDAC [32,33]. PDAC is characterized by profound desmoplasia, impaired perfusion, thrombosis, and hypoxia, all of which promote aggressive growth, invasion, immune evasion, and resistance to treatment [34]. In preclinical PDAC models, exogenous intratumoral PS reduced tumor volume and mass, reduced intratumoral hypoxia, and improved thrombin-generation parameters [35]. Consistent with these observations, our prior cBioPortal-based analysis demonstrated that high intratumoral *PROS1* expression was associated with improved survival in PDAC and reduced expression of genes linked to oncogenic growth, hypoxia, invasion, metastasis, and EMT [33]. Importantly, high *PROS1* expression was associated with reduced expression of *CA9*, a canonical hypoxia-inducible factor-1 (HIF-1) target that supports tumor adaptation to hypoxia and extracellular acidosis [20].

Although hypoxia can be detrimental to normal tissue, in PDAC, it drives tumor survival and progression [36]. Hypoxia-inducible factors coordinate signaling pathways that support metabolic adaptation, angiogenesis, invasion, therapy resistance, and immune suppression [36,37]. Experimental evidence indicates that hypoxia suppresses PS expression, whereas PS administration can attenuate hypoxia-associated signaling in PDAC models [20,35]. This reciprocal relationship raises the possibility that reduced intratumoral *PROS1* expression may facilitate a hypoxia-associated, pro-tumor state, while higher *PROS1* expression may be associated with a less aggressive tumor microenvironment.

Tumor evolution is also shaped by genomic instability, which can promote intratumoral heterogeneity, clonal selection, therapy resistance, and metastatic competence [38,39,40]. However, genomic instability is not a single biological process and cannot be adequately represented by one measurement alone. We therefore evaluated three complementary measures: tumor mutational burden (TMB), aneuploidy score (AS), and tumor break load (TBL), where available [41].

Tumor mutational burden quantifies somatic mutations per megabase of sequenced DNA and reflects the burden of nucleotide-level genomic alterations. High TMB may increase neoantigen generation and tumor immunogenicity, although its biological and clinical implications vary substantially across tumor types [42,43]. In contrast, the aneuploidy score reflects chromosome-scale copy-number abnormalities and provides a measure of chromosomal imbalance [41,44]. Tumor break load (TBL) provides an additional structural measure of chromosomal instability by quantifying unbalanced somatic structural-variant-associated breakpoints, thereby capturing chromosomal breakage events underlying structural rearrangements [41,45].

Simultaneous assessment of TMB, aneuploidy score, and TBL is therefore biologically informative because these measures interrogate distinct but complementary dimensions of genomic instability. Evaluating these complementary features enables us to determine whether associations between *PROS1* expression and survival are accompanied by a broader genomic-instability phenotype rather than reflecting only one class of genomic alteration. This approach is particularly relevant because cell-cycle dysregulation, defective DNA-damage responses, replication stress, hypoxia, and chromosomal instability represent interconnected processes that can promote genomic instability and malignant progression [46,47,48,49,50].

The objective of this study was to evaluate the association of intratumoral *PROS1* expression with overall survival, hypoxia-associated gene expression, and multiple dimensions of genomic instability across nine cancer types for which *PROS1*-dependent tumor biology has not been comprehensively characterized. Using publicly available cBioPortal datasets, we analyzed bladder urothelial carcinoma, cervical squamous cell carcinoma, esophageal adenocarcinoma, lung adenocarcinoma, lung squamous cell carcinoma, ovarian serous cystadenocarcinoma, clear-cell renal cell carcinoma, non-clear-cell renal carcinoma, and stomach adenocarcinoma. Tumors were stratified by low versus high *PROS1* expression, and associations with survival, TMB, aneuploidy score, TBL, and hypoxia-related markers were examined.

We further performed meta-analyses of selected genes involved in oncogenic signaling, cell-cycle regulation, hypoxia, and EMT in cancer types that demonstrated survival and genomic-instability patterns resembling those observed in PDAC [33]. We hypothesized that high intratumoral *PROS1* expression would identify tumor subsets with improved survival, reduced hypoxia-associated signaling, and lower genomic instability. By defining the tumor types in which this pattern is most evident, this exploratory analysis provides a prioritized framework for mechanistic and in vivo studies of PS as a regulator of tumor progression.

## 2. Results

### 2.1. Association of Intratumoral PROS1 Expression with Survival Differs Across Cancer Types

*PROS1* expression has been associated with context-dependent effects on tumor biology. For example, *PROS1* has been associated with tumor-suppressive activity in breast cancer [23], lung cancer [24], and PDAC [33,35]; whereas functional studies in glioblastoma and oral squamous cell carcinoma support tumor-promoting roles [31,51]. In uveal melanoma, *PROS1* may additionally contribute to tumor progression indirectly by promoting an immunosuppressive microenvironment through MERTK signaling in macrophages [29]. These findings underscore the context-dependent nature of *PROS1* [52]. We first examined the association between intratumoral *PROS1* expression and overall survival across nine cancer types. Tumors were stratified into high- and low-*PROS1*-expression groups as described in the Methods. The direction of the survival association varied by tumor type, indicating that the clinical relevance of *PROS1* expression is context-dependent.

High intratumoral *PROS1* expression was associated with poorer overall survival in bladder urothelial carcinoma, cervical squamous cell carcinoma, esophageal adenocarcinoma, lung squamous cell carcinoma, non-clear-cell renal carcinoma, and stomach adenocarcinoma (Table 1). In contrast, high *PROS1* expression was associated with improved overall survival in lung adenocarcinoma, ovarian serous cystadenocarcinoma, and clear-cell renal cell carcinoma. The strongest adverse association was observed in non-clear-cell renal carcinoma (pooled hazard ratio [HR], 2.027; 95% confidence interval [CI], 1.778–2.275; *p* = 4.4 × 10^−3^), whereas the greatest survival benefit associated with high *PROS1* expression was observed in clear-cell renal cell carcinoma (pooled HR, 0.675; 95% CI, 0.553–0.797; *p* = 9.2 × 10^−4^).

Lung adenocarcinoma (pooled HR, 0.679; 95% CI: 0.553–0.805; *p* = 2.2 × 10^−3^) and ovarian serous cystadenocarcinoma (pooled HR, 0.681; 95% CI: 0.557–0.804; *p* = 1.5 × 10^−3^) demonstrated survival patterns comparable to those previously identified in pancreatic ductal adenocarcinoma (PDAC) [33]. These tumor types were therefore prioritized for subsequent analysis of genomic instability, hypoxia-associated signatures, and *PROS1*-dependent correlation patterns in oncogenic, cell-cycle, EMT, and extracellular-matrix-associated genes. Note that survival analyses were conducted via exploratory, unadjusted Mantel-Haenszel HR analyses, and the independent prognostic value of *PROS1* remains to be established in PDAC, lung adenocarcinoma, and ovarian serous cystadenocarcinoma.

When *PROS1* expression was modeled as a continuous variable, no statistically significant association with overall survival was detected in the evaluated cohorts (Supplementary Table S1). In Cox proportional hazards regression, a hazard ratio (HR) of 1 indicates no association between the predictor and the hazard of the event, whereas HRs above or below 1 indicate higher or lower hazards, respectively [33,53]. All estimated HRs per unit increase in *PROS1* expression were close to 1 and not statistically significant and therefore, do not provide sufficient evidence of a meaningful linear association between *PROS1* expression and overall survival. Accordingly, the survival differences observed in the dichotomized analyses should not be interpreted as evidence of a consistent linear dose–response relationship across the full range of *PROS1* expression. Thus, the survival differences observed after dichotomization of *PROS1* expression may reflect a threshold-dependent or otherwise non-linear association rather than a uniform dose–response relationship across the full expression range.

### 2.2. High PROS1 Expression Is Associated with Reduced Genomic Instability Metrics in Selected Cancer Types

As described above, we incorporated three complementary measures of genomic instability: tumor mutational burden (TMB), tumor break load (TBL), and aneuploidy score (AS), reflecting the density of somatic mutations, structural-variant-associated chromosomal breakage, and the extent of chromosome-scale copy-number gains and losses, respectively. Joint evaluation of these measures enabled us to determine whether *PROS1* expression was associated with a specific class of genomic alteration or with a broader genomic-instability phenotype; AS captures the proportion of the genome affected by copy-number alterations and therefore reflects broad chromosomal copy-number disruption.

PDAC was included in this analysis because, although *PROS1* has recently been investigated in relation to tumor progression and survival in PDAC [33], its relationship with chromosomal instability has not been specifically characterized. Thus, evaluating PDAC alongside other tumor types allowed us to determine whether the association between *PROS1* expression and genomic instability extends beyond the molecular and clinical features previously investigated in this disease.

Microsatellite-instability metrics correlations with *PROS1* expression were either not statistically significant or were of negligible magnitude across the evaluated cohorts. Pooled Pearson R values, confidence intervals, and *p*-values of MSI results are shown in Supplementary Table S2. These results are therefore not interpreted further.

#### 2.2.1. Tumor Mutational Burden (TMB)

Tumor mutational burden (TMB) is the number of genetic changes in cancer cell DNA and acts as a biomarker for how well a patient would respond to immunotherapy. Tumor mutational burden is conventionally expressed as the number of somatic mutations per megabase of sequenced DNA, as reported in cBioPortal [54]. High *PROS1* expression was inversely correlated with TMB in bladder urothelial carcinoma, cervical squamous cell carcinoma, esophageal adenocarcinoma, lung adenocarcinoma, lung squamous cell carcinoma, ovarian serous cystadenocarcinoma, pancreatic ductal adenocarcinoma (PDAC), and stomach adenocarcinoma. Correlation magnitudes were generally weak to moderate, consistent with the multifactorial nature of somatic mutational burden (Table 2).

The most pronounced inverse relationship was observed in PDAC (*r* = −0.429; 95% CI, −0.474 to −0.329; *p* = 1.0 × 10^−4^). Among the newly evaluated tumor types, lung adenocarcinoma demonstrated the strongest inverse association (*r* = −0.231; 95% CI, −0.302 to −0.150; *p* = 1.6 × 10^−6^), followed by ovarian serous cystadenocarcinoma (*r* = −0.204; 95% CI, −0.326 to −0.075; *p* = 2.2 × 10^−3^). No pooled correlation between *PROS1* expression and TMB was observed in clear-cell or non-clear-cell renal carcinoma, since correlation coefficients are close to zero (Table 2).

These findings indicate that high intratumoral *PROS1* expression identifies tumor subsets with lower mutational burden in selected malignancies. However, this association should not be interpreted as evidence that *PROS1* directly suppresses mutation acquisition.

#### 2.2.2. Tumor Break-Load (TBL) Score

Additionally, TBL score was assessed to determine whether *PROS1* expression was also associated with structural chromosomal instability. A high TBL score is associated with significant changes in gene expression in 26/31 cancer types. This score quantifies unbalanced structural-variant-associated chromosomal breakpoints and is therefore conceptually distinct from TMB. High TBL has been associated with transcriptional programs involving DNA-damage response and diminished immune activity and has been linked to worse clinical outcomes in several cancer types [41].

An inverse association between *PROS1* expression and TBL was observed in bladder urothelial carcinoma, lung squamous cell carcinoma, ovarian serous cystadenocarcinoma, and PDAC. The strongest correlation was again observed in PDAC (*r* = −0.622; 95% CI, −0.634 to −0.459; *p* = 1.0 × 10^−4^). Among the newly examined tumor types, ovarian serous cystadenocarcinoma showed the most pronounced inverse association (*r* = −0.208; 95% CI, −0.317 to −0.093; *p* = 5.0 × 10^−4^) (Table 3).

In contrast, clear-cell renal cell carcinoma, non-clear-cell renal carcinoma, and stomach adenocarcinoma exhibited weak positive correlations between *PROS1* expression and TBL. TBL data were unavailable for cervical squamous cell carcinoma, esophageal adenocarcinoma, and lung adenocarcinoma in the analyzed cBioPortal datasets Table 3). These results further support a tumor-type-specific association between *PROS1* expression and structural genomic instability.

#### 2.2.3. Aneuploidy Score (AS)

We next examined the association between *PROS1* expression and aneuploidy score (AS), which quantifies the total number of chromosomal arm-level gains and losses in a tumor adjusted for ploidy. Higher values indicate more extensive chromosome-scale copy-number abnormalities. Aneuploidy-associated copy-number alteration is distinct from point-mutation burden and has been associated with tumor evolution, treatment resistance, and clinical outcome [55].

As shown in Table 4, higher *PROS1* expression was inversely correlated with AS in bladder urothelial carcinoma, cervical squamous cell carcinoma, esophageal adenocarcinoma, lung adenocarcinoma, lung squamous cell carcinoma, ovarian serous cystadenocarcinoma, PDAC, and stomach adenocarcinoma. Lung adenocarcinoma exhibited the strongest inverse association among the newly analyzed cancers (*r* = −0.241; 95% CI, −0.329 to −0.149; *p* = 5.8 × 10^−3^), whereas PDAC again exhibited the strongest overall association (*r* = −0.581; 95% CI, −0.621 to −0.410; *p* = 1.0 × 10^−4^).

Clear-cell and non-clear-cell renal carcinomas were exceptions, showing weak positive associations between *PROS1* expression and AS. Together with the TMB and TBL findings, these results suggest that the association between high *PROS1* expression and lower genomic-instability metrics is most consistent in PDAC, lung adenocarcinoma, and ovarian serous cystadenocarcinoma, but is not conserved across all tumor types (Table 4).

### 2.3. PROS1 Expression Is Inversely Associated with Hypoxia Signatures in Selected Cancer Types

We next examined whether *PROS1* expression was associated with three established transcriptome-based hypoxia signatures: the Buffa, Winter, and Ragnum hypoxia scores. These signatures summarize expression patterns induced by tumor hypoxia. The Ragnum Hypoxia score assigns positive values to highly hypoxic states, while a score of 0 indicates baseline hypoxia. For the Buffa and Winter scores, higher values indicate a more hypoxic transcriptional state [56,57].

Higher *PROS1* expression was inversely correlated with the Buffa and Winter hypoxia scores in bladder urothelial carcinoma, cervical squamous cell carcinoma, lung adenocarcinoma, lung squamous cell carcinoma, ovarian serous cystadenocarcinoma, PDAC, and non-clear-cell renal carcinoma. The strongest and most consistent inverse correlations were observed in lung adenocarcinoma, ovarian serous cystadenocarcinoma, and PDAC. In lung adenocarcinoma, *PROS1* expression was inversely associated with the Buffa (*r* = −0.318), Winter (*r* = −0.304), and Ragnum (*r* = −0.316) hypoxia scores (all *p* = 1.0 × 10^−4^). Similar relationships were observed in ovarian serous cystadenocarcinoma and PDAC (Table 5).

Clear-cell renal cell carcinoma differed from the remaining tumor types, exhibiting positive correlations between *PROS1* expression and all three hypoxia scores. This divergent pattern is consistent with the context-dependent survival and genomic-instability associations observed in renal tumors and suggests that the relationship between *PROS1* and hypoxia-related signaling is not uniform across cancer lineages.

Esophageal adenocarcinoma and stomach adenocarcinoma were not evaluated because these hypoxia-signature data were unavailable in the analyzed datasets. Overall, these data identify lung adenocarcinoma and ovarian serous cystadenocarcinoma as the tumor types most closely recapitulating the inverse association between *PROS1* expression and hypoxia-associated transcriptional programs previously observed in PDAC.

### 2.4. PROS1-Associated Correlation Shifts Identify Convergent Signaling Patterns in Lung Adenocarcinoma, Ovarian Serous Cystadenocarcinoma, and PDAC

Based on the integrated survival, genomic-instability, and hypoxia analyses, lung adenocarcinoma and ovarian serous cystadenocarcinoma were selected for further comparison with PDAC. These tumor types satisfied three prespecified criteria: (i) improved survival in the high-*PROS1* group; (ii) an inverse correlation between *PROS1* expression and at least one hypoxia metric of magnitude *r* < −0.200; and (iii) an inverse correlation between *PROS1* expression and at least one genomic-instability metric of magnitude *r* < −0.200.

We then examined whether the relationships between *PROS1* and selected genes differed between low- and high-*PROS1*-expression tumor subsets. For each gene, we calculated Δ*R* as the difference between the Pearson correlation coefficient in the high-*PROS1* group and that in the low-*PROS1* group:

Δ*R* = *R*_high PROS1_ − *R*_low PROS1_

A negative Δ*R* indicates that the correlation became more negative, or shifted from positive to negative, in tumors with high *PROS1* expression. This analysis was designed to identify genes whose co-expression relationship with *PROS1* changed substantially according to the *PROS1* expression state.

Only genes that met all prespecified interpretability criteria were included in pooled analyses: (i) a statistically significant correlation in at least one *PROS1* stratum; (ii) a meaningful shift in correlation magnitude between low- and high-*PROS1* groups; and (iii) concordant direction and broadly similar effect magnitude across contributing cohorts. Genes with discordant correlation direction between cohorts or negligible correlation shifts were excluded because a pooled estimate would not provide a biologically interpretable summary. Full gene-level correlation results are provided in Supplementary Tables S3–S5, and cohort heterogeneity analyses are presented in Supplementary Tables S6–S8.

Across eligible genes, heterogeneity was generally low to moderate. Cochran’s Q statistic, its associated *p*-value, and the *I*^2^ statistic were used to assess between-cohort variation. Most genes showed no statistically significant evidence of heterogeneity, supporting the use of pooled estimates.

Between-cohort heterogeneity was evaluated with Cochran’s Q statistic, Q *p*-value, and the *I*^2^ metric for each gene. For all three cancers, all Q tests were nonsignificant (*p* < 0.05), indicating that no variability exists beyond expected sampling error. Across all analyzed cohorts (4 in PDAC, 3 in NSCLC lung adenocarcinoma, and 2 in ovarian serous cystadenocarcinoma) that demonstrated significant and appropriate magnitude ΔR values, only 17 genes had Q values exceeding degrees of freedom (df = 3 for PDAC, df = 2 for lung adenocarcinoma, and df = 1 for ovarian serous cystadenocarcinoma), and the *I*^2^ values exceeded 0.10%. This is consistent with low-to-moderate heterogeneity by conventional thresholds across multiple cohorts (Supplementary Tables S6–S8).

Across lung adenocarcinoma (Table 6), ovarian serous cystadenocarcinoma (Table 7), and PDAC (Table 8), *ANAPC2* displayed a significant negative shift in correlation between low- and high-*PROS1* tumor subsets. This shared pattern links high *PROS1* expression to altered coordination of cell-cycle-regulatory signaling, because *ANAPC2* encodes a core component of the anaphase-promoting complex/cyclosome (APC/C), an E3 ubiquitin ligase that regulates mitotic progression [58].

Lung adenocarcinoma and ovarian serous cystadenocarcinoma also demonstrated pronounced negative correlation shifts for *SNAI2* and *MMP9*, consistent with altered EMT- and extracellular-matrix-remodeling-associated transcriptional signaling in high-*PROS1* tumors. In lung adenocarcinoma, there was an observed correlation shift in *MMP2*. In ovarian serous cystadenocarcinoma, *SLC2A1*, *MMP7*, and *SNAI1* showed negative shifts, linking high-*PROS1* expression to altered hypoxia/metabolic adaptation and EMT-associated gene regulation [59,60,61]. *SLC2A1* encodes glucose transporter 1 (GLUT1), a HIF-responsive transporter frequently induced under hypoxic conditions [59].

PDAC showed correlation shifts in multiple cell-cycle-associated genes, including *KNL1*, *AURKC*, *ANAPC2*, *CAMSAP3*, and *ANAPC4*. The strongest shift was observed for *ANAPC4* (Δ*R* = −0.704), further supporting the association between high *PROS1* expression and altered cell-cycle-regulatory transcriptional relationships in PDAC [62]. Collectively, these findings identify convergent *PROS1*-associated alterations in cell-cycle regulation, EMT, extracellular-matrix remodeling, and hypoxia-associated metabolic signaling across lung adenocarcinoma, ovarian serous cystadenocarcinoma, and PDAC.

Importantly, these analyses identify correlation-pattern shifts rather than direct transcriptional regulation. Therefore, the findings should be interpreted as evidence of tumor-subtype-specific co-expression networks associated with *PROS1* expression, which require experimental validation to establish causal mechanisms.

## 3. Discussion

This cross-cancer analysis demonstrates that the association between intratumoral *PROS1* expression and clinical outcome is strongly tumor-context dependent. High *PROS1* expression in exploratory, unadjusted Mantel-Haenszel HR analyses was associated with poorer overall survival in bladder urothelial carcinoma, cervical squamous cell carcinoma, esophageal adenocarcinoma, lung squamous cell carcinoma, non-clear-cell renal carcinoma, and stomach adenocarcinoma. In contrast, high *PROS1* expression was associated with improved survival in lung adenocarcinoma, ovarian serous cystadenocarcinoma, and clear-cell renal cell carcinoma. These findings extend our previous observations in pancreatic ductal adenocarcinoma (PDAC), in which high intratumoral *PROS1* expression was associated with improved survival and transcriptional patterns consistent with reduced hypoxia, oncogenic signaling, and epithelial–mesenchymal transition (EMT). Together, the results indicate that *PROS1* is unlikely to function as a universal tumor suppressor or oncogene. Instead, its clinical significance appears to depend on tumor lineage, genomic context, cellular composition, microenvironmental conditions, and the signaling networks active within individual malignancies.

The present study was designed to determine whether the favorable survival association observed in PDAC was accompanied by a broader molecular phenotype in other tumor types. We therefore evaluated tumor mutational burden (TMB), tumor break load (TBL) score, and aneuploidy score (AS) as complementary measures of genomic instability. These metrics capture distinct, nonredundant features of tumor genome disruption. Their joint analysis provides a more rigorous assessment of genomic instability than any individual metric alone.

Across the analyzed cohorts, high *PROS1* expression was generally associated with lower TMB, TBL score, and/or AS, although the direction and strength of these associations differed by cancer type. The most consistent inverse relationships were observed in PDAC, lung adenocarcinoma, and ovarian serous cystadenocarcinoma. In PDAC, high *PROS1* expression was inversely associated with all three genomic-instability measures, including TMB, TBL score, and AS. Lung adenocarcinoma demonstrated inverse associations with TMB and AS, whereas ovarian serous cystadenocarcinoma demonstrated inverse associations with TMB, TBL score, and AS. These findings suggest that high intratumoral *PROS1* expression identifies PDAC, lung adenocarcinoma, and ovarian serous cystadenocarcinoma characterized by lower somatic mutation burden, reduced structural chromosomal disruption, and/or fewer chromosome-arm-level copy-number alterations.

However, this interpretation should remain associative rather than causal. The present analysis does not establish that PS directly maintains genomic stability or prevents mutational or chromosomal damage. Rather, *PROS1* expression may mark tumors with a less genomically unstable evolutionary state, a distinct tumor-cell lineage, altered stromal or immune composition, or microenvironmental conditions that are less permissive to DNA damage, replication stress, chromosomal instability, and aggressive clonal selection. Conversely, low *PROS1* expression may identify tumors in which hypoxia, hypercoagulability, altered vascular function, and genomic instability coexist. Functional studies using genetic and pharmacologic manipulation of PS will be required to distinguish these possibilities.

The association between *PROS1* expression and hypoxia-associated transcriptional programs further supports this model. High *PROS1* expression was inversely associated with the Buffa and Winter hypoxia scores in most evaluable cancer types and with all three hypoxia scores in lung adenocarcinoma, ovarian serous cystadenocarcinoma, and PDAC. These three tumor types therefore demonstrated a convergent pattern of improved survival, lower genomic-instability metrics, and reduced hypoxia-associated transcriptional activity in high-*PROS1* tumors. This pattern is notable because hypoxia can promote malignant progression by altering cellular metabolism, stimulating angiogenic and invasive programs, impairing DNA repair, increasing replication stress, selecting for aggressive clones, and limiting anti-tumor immune activity [49,63]. In PDAC specifically, hypoxia is a prevalent feature of the poorly perfused, desmoplastic microenvironment and has been linked to therapeutic resistance and poor clinical outcome [64,65].

The hypoxia findings are also consistent with preclinical evidence that exogenous Protein S (PS) can reduce intratumoral hypoxia in PDAC xenografts. In this study, intratumoral PS administration reduced PDAC tumor volume and mass, decreased CA9 expression as a marker of intratumoral hypoxia, reduced angiogenic and proliferative markers, and increased apoptotic activity [35]. These findings provide biological plausibility for a potential PS–coagulation–tumor microenvironment–hypoxia axis in PDAC. However, the current cross-cancer findings do not establish that the same mechanism operates in lung adenocarcinoma or ovarian serous cystadenocarcinoma. The observed inverse correlations may instead reflect tumor-cell-intrinsic PROS1 biology, contributions from stromal or endothelial cells, differences in immune-cell composition, or a combination of these factors.

Clear-cell renal cell carcinoma was a notable exception. Although high *PROS1* expression was associated with improved survival in this tumor type, it was positively associated with TBL score, AS, and hypoxia-signature scores. This discordance emphasizes that survival associations should not be interpreted in isolation and reinforces the context dependence of *PROS1*-associated signaling. Clear-cell renal cell carcinoma has a distinctive biology characterized by frequent dysregulation of oxygen-sensing pathways, including VHL–HIF signaling [66]. Accordingly, transcriptome-based hypoxia scores and chromosome-level alterations may have different biological implications in clear-cell renal cell carcinoma than in PDAC, lung adenocarcinoma, or ovarian serous cystadenocarcinoma. Clear-cell renal cell carcinoma should therefore be considered a mechanistically distinct subgroup requiring separate investigation rather than being incorporated into a common PS-associated anti-hypoxia model.

Lung adenocarcinoma and ovarian serous cystadenocarcinoma were prioritized for targeted gene-correlation analyses because they most closely reproduced the PDAC-associated pattern: improved survival in the high-*PROS1* subgroup, an inverse association between *PROS1* expression and at least one hypoxia score, and an inverse association with at least one genomic-instability metric. Stratification by low versus high *PROS1* expression revealed substantial shifts in the correlations between *PROS1* and selected genes involved in cell-cycle control, EMT, extracellular-matrix remodeling, and hypoxia-associated metabolic adaptation. These correlation shifts indicate that *PROS1* is embedded in different transcriptional networks in tumors with low versus high *PROS1* expression. They do not establish direct transcriptional regulation by *PROS1* or PS.

A shared finding across lung adenocarcinoma, ovarian serous cystadenocarcinoma, and PDAC was the altered *PROS1*–*ANAPC2* correlation in high-*PROS1* tumors. *ANAPC2* encodes a core subunit of the anaphase-promoting complex/cyclosome (APC/C), an E3 ubiquitin ligase that regulates mitotic progression through the controlled degradation of key cell-cycle regulators, including securin and cyclins [67,68,69]. Dysregulation of APC/C activity or mitotic checkpoint control can promote chromosome missegregation, aneuploidy, and chromosomal instability, processes that contribute to tumor evolution [70,71]. Thus, the altered *PROS1–ANAPC2* relationship observed is intriguing in the context of the genomic-instability phenotype. However, these correlation shifts do not establish that Protein S directly regulates APC/C function; mechanistic studies will be required to determine whether PS influences cell-cycle progression, mitotic checkpoints, DNA-damage responses, or chromosome segregation.

The EMT-associated transcription factor *SNAI2* (Slug) and the matrix-remodeling gene *MMP9* also showed substantial negative correlation shifts in both lung adenocarcinoma and ovarian serous cystadenocarcinoma. *SNAI2* is a well-established regulator of EMT, invasion, and cancer-cell plasticity [33]. It also has a cell-cycle-dependent role in genome maintenance. At the G1/S transition, cyclin E–CDK2 phosphorylates Slug at Ser54 and Ser104, promoting ubiquitination and proteasomal degradation. Stabilization of nonphosphorylatable Slug impairs the expression of DNA-synthesis and checkpoint-associated proteins, delays S-phase progression, and contributes to genomic instability [72]. Thus, the relationship between *SNAI2*, cell-cycle control, and genomic stability is complex and cannot be reduced to the simple premise that lower Slug expression necessarily indicates greater or lesser genomic stability.

Importantly, *SNAI2* and *ANAPC2* should not be interpreted as components of a proven linear *PROS1*–Slug–APC/C signaling pathway. Instead, they represent two biologically relevant but distinct dimensions of the tumor phenotype identified in the present analysis. *SNAI2* provides a link to EMT, invasive plasticity, stress adaptation, and therapy resistance, whereas *ANAPC2* provides a link to APC/C-dependent cell-cycle control, mitotic exit, and chromosome-segregation biology. Their shared *PROS1*-associated correlation shifts may therefore reflect a broader transcriptional state in which invasive plasticity and mitotic-control programs are differently coordinated in high-*PROS1* and low-*PROS1* tumors. Future functional studies should determine whether Protein S directly modifies hypoxia signaling, cell-cycle progression, checkpoint integrity, APC/C activity, chromosomal segregation, EMT plasticity, or treatment response in ovarian carcinoma models.

The *MMP2*, *MMP7*, and *MMP9* findings further implicate extracellular matrix remodeling and invasion-associated signaling [73]. In lung adenocarcinoma, *MMP2* showed a significant correlation shift, while *MMP7* and *MMP9* showed significant shifts in ovarian serous cystadenocarcinoma. *MMP9* was altered in both tumor types. These metalloproteinases influence extracellular matrix turnover, tumor–stroma interactions, invasion, angiogenesis, and metastatic dissemination [73]. Their changed correlation patterns in high-*PROS1* tumors are therefore compatible with altered invasive and microenvironmental signaling. However, these associations do not establish decreased metalloproteinase activity, reduced invasion, or a direct PS-dependent effect. Alteration of *MMP2*, *MMP7*, and *MMP9* correlation patterns also reflects that further study is needed on the potential impact of *PROS1* on ECM composition, immune signaling in the tumor microenvironment, and cancer cell migration [74,75].

Ovarian serous cystadenocarcinoma also demonstrated a marked correlation shift in *PROS1*–*SLC2A1*. *SLC2A1* encodes glucose transporter 1 (GLUT1), which supports glucose uptake and metabolic adaptation in hypoxic tumors [59]. The negative correlation observed in high-*PROS1* tumors is consistent with the parallel inverse associations between *PROS1* expression and transcriptome-based hypoxia scores in ovarian serous cystadenocarcinoma. This convergence supports the hypothesis that high *PROS1* expression marks a tumor state with diminished hypoxia-associated metabolic adaptation. However, it remains necessary to determine whether PS directly alters oxygenation, HIF signaling, glucose uptake, or the cellular composition of the tumor microenvironment.

PDAC displayed additional correlation shifts involving *KNL1*, *AURKC*, *CAMSAP3*, and *ANAPC4*. These genes participate in mitotic-spindle assembly, kinetochore function, chromosome segregation, microtubule organization, and APC/C-mediated cell-cycle progression [67,76,77,78]. The particularly strong negative shift involving *ANAPC4* is consistent with a *PROS1*-associated transcriptional state linked to cell-cycle and chromosome-segregation programs. Given the strong inverse associations between *PROS1* expression and TMB, TBL, AS, and hypoxia scores in PDAC, these observations provide a plausible molecular framework for future experiments. Differences in tumor-suppressor gene alterations, tumor microenvironment composition, and tissue architecture may contribute to the distinct molecular profiles observed between high- and low-*PROS1*-expressing PDAC tumors. Investigating these factors may help elucidate the biological mechanisms underlying the association between *PROS1* expression and patient outcomes and clarify its potential utility as a prognostic biomarker for identifying molecularly distinct PDAC subgroups. Appropriate studies could include PS supplementation, *PROS1* knockdown or overexpression, and assessment of HIF signaling, DNA-damage markers, replication stress, checkpoint integrity, mitotic errors, copy-number alteration, and tumor growth in relevant PDAC, lung adenocarcinoma, and ovarian carcinoma models.

### Limitations

This study has several limitations. First, all analyses relied on retrospective, publicly available datasets, which differ in sample acquisition, tissue handling, sequencing platform, read depth, bioinformatic processing, clinical annotation, and cohort composition. Such heterogeneity can affect expression estimates, genomic-instability metrics, survival analyses, and pooled effect sizes. Although we evaluated inter-cohort heterogeneity for selected gene-correlation analyses, residual technical and biological heterogeneity cannot be excluded.

Second, the available clinical data were incomplete and inconsistently reported. In particular, treatment regimen, treatment timing, stage, grade, performance status, race and ethnicity, smoking history where relevant, molecular subtype, and other prognostic variables were unavailable or incompletely annotated in some cohorts. We were therefore unable to determine whether the observed survival associations were independent of these potential confounders. The high-*PROS1* and low-*PROS1* survival differences should consequently be regarded as unadjusted or incompletely adjusted associations unless multivariable Cox regression was performed and reported. The impact of *PROS1* on cancer prognosis necessitates further evaluation and cannot be concluded by the above analyses.

Third, bulk RNA-sequencing data do not establish the cellular source of *PROS1* expression. Intratumoral *PROS1* may arise from malignant epithelial cells, endothelial cells, fibroblasts, macrophages, other immune populations, or mixtures of these compartments. Therefore, observed associations may reflect tumor-cell-intrinsic biology, microenvironmental composition, tumor purity, vascularity, inflammatory state, or stromal abundance. Single-cell RNA sequencing, spatial transcriptomics, immunohistochemistry, and protein-level validation are needed to localize PS expression and define the relevant cellular and spatial context.

Fourth, the study identifies associations rather than causality. The analyses cannot establish that PS regulates hypoxia, genomic instability, EMT, extracellular-matrix remodeling, cell-cycle progression, or survival. Likewise, the differential correlation analyses define changes in co-expression patterns; they do not demonstrate direct regulatory relationships between *PROS1* and *ANAPC2*, *SNAI2*, *MMP9*, *SLC2A1*, or other evaluated genes. Due to the exploratory nature of the study, changes in the low- and high-*PROS1* expression effects affected by stratification, range restriction, and non-linear relationships were not included, and as such, all associations analyzed remain hypothesis-generating.

Fifth, TMB, TBL, and AS quantify distinct aspects of genomic instability but are influenced by tumor purity, sequencing methodology, coverage, copy-number calling, variant filtering, and the specific dataset from which they were obtained. Missing availability of TBL, TMB, or hypoxia-score data in individual cohorts limited direct comparison across all cancer types. TMB should also not be interpreted as a universal predictor of immune-checkpoint-inhibitor response, because its predictive performance varies across tumor types, and clinically relevant thresholds may differ according to tumor context, assay platform, and sequencing methodology [54].

Finally, the number of analyses performed across cancer types, genomic-instability metrics, hypoxia signatures, and selected genes creates a risk of false-positive findings. Unless a formal multiple-testing correction was applied, nominal *p*-values should be interpreted as purely exploratory. Independent replication in external cohorts, preferably with prespecified thresholds and false-discovery-rate correction, is essential before clinical translation.

## 4. Materials and Methods

### 4.1. Cbioportal Cohorts and Cohort Selection

Cohorts with RNA-sequencing data and associated survival and genomic instability data were selected from cBioPortal for Cancer Genomics (https://www.cbioportal.org, accessed on 1 June 2026) [79,80,81]. RNA-sequencing data generated from Illumina HiSeq were obtained from The Cancer Genome Atlas (TCGA) through cBioPortal.

To ensure the independence of observations in pooled analyses, patient identifiers were reviewed across all datasets to identify potential patient overlap. Patients appearing in multiple datasets were identified, and duplicate observations were removed to ensure that each patient contributed only once to the pooled analyses.

Due to previously published data on *PROS1* signaling and/or survival, the following cancers were not included in the current analysis: breast carcinoma [23], glioblastoma [25], prostate carcinoma [26], hepatocellular carcinoma [27], thyroid cancer [23], endometrial carcinoma [28], uveal melanoma [29], sarcoma [30], and squamous head and neck cancer [31]. Analysis of colorectal adenocarcinoma was excluded because only one cohort contained RNA-sequencing data, survival, and genomic instability data.

Furthermore, cancer types with insufficient sample sizes to achieve sufficient study power were not evaluated. These cancers included the following: testicular germ cell tumors, thymomas, cholangiocarcinoma, uterine carcinoma/mixed Mullerian tumors, adrenocortical carcinoma, and kidney chromophobe cancer.

Hence, cancer types with sufficient sample size, survival, and genomic instability data were selected. Note that not all cancers had complete *PROS1* data or data for certain parameters, and data were pooled when available. Overall, the following cohorts were evaluated with the selected number of patients (based on available *PROS1* data) (Table 9):

Additional batch correction was not applied since transcriptomic datasets were not integrated. Furthermore, dichotomized analyses were conducted within cohort rather than with pooled transcriptomic data to limit cross-study bias. Only standardized summary measures were generated and pooled across cohorts for each cancer type where data were available for comparative purposes.

### 4.2. Dichotomization of High and Low Intratumoral Pros1 Expression

To minimize the potential influence of extreme survival times on the survival analysis, the upper and lower 5% of the observed survival-time distribution were excluded prior to analysis. This trimming procedure was used as a conservative analytical approach to limit the influence of observations at distributional extremes while retaining the vast majority of the study population. The 5% threshold was selected as an operational criterion for identifying extreme observations and preventing end result bias [82].

High *PROS1* expression was defined as samples greater than or equal to the median expression, and low *PROS1* was defined as samples less than the cohort median. A median split is justified when dichotomizing a continuous variable and is useful when data were not normally distributed in each cohort [83].

Additionally, Cox regression analysis was performed to model *PROS1* expression as a continuous variable in each cancer cohort to determine if the survival association reflects a dose-response relationship. Hazard ratios with 95% confidence intervals were calculated.

### 4.3. Hazard Ratio Pooling: Survival of Patients with Low v. High Intratumoral Pros1 Expression

From all cohorts, the top and bottom 5% of outliers were removed from survival data to prevent data skewing. The reported Mantel-Haenszel hazard ratio and 95% confidence interval were transformed to natural logarithms, and the log-transformed standard error of the log-transformed hazard ratio (assuming a 95% confidence interval was derived with the formula $\mathrm{SE}=\frac{\ln(\text{Upper CI})-\ln(\text{Lower CI})}{2\times 1.96}$. We calculated pooled effect estimates on the log-HR scale using an inverse-variance fixed-effect model that accounts for sample size. We then exponentiated the pooled log-values to obtain the pooled HR and 95% CI. The two-tailed *p*-values were pooled using a weighted method of pooling, Stouffer’s Z method. All analyses were performed in Microsoft Excel 2024 and GraphPad Prism v.10.

### 4.4. Pooling of Correlation Coefficients, Confidence Intervals and p-Values for Genomic Instability Markers, Hypoxia Markers, and Gene Expression Changes

Pearson correlation coefficients (R) were generated for *PROS1* expression against genomic instability markers TMB, TBL-score, and AS, and in comparing *PROS1* expression to Ragnum, Winter, and Buffa hypoxia scores, where data were available, for all cancers. Pearson correlation coefficients (R) were also generated in comparison of gene expression under high and low *PROS1* expression conditions for all cohorts of lung adenocarcinoma and ovarian serous cystadenocarcinoma. Pearson R correlation coefficients were implemented due to normal data distribution.

When applicable, two or more Pearson R correlation coefficients for the same metric for the same cohort were pooled. First, correlation coefficients were transformed to Fisher’s z′ values using the formula $z^{\prime }=\frac{1}{2}\ln\left(\frac{1+r}{1-r}\right)$, and z′ values were pooled with inverse-variance weighting, where weights were defined as sample size (n) − 3. From there, pooled Fisher’s z′ values were converted to a Pearson correlation coefficient with the inverse transformation $r=\frac{e^{2z^{\prime }}-1}{e^{2z^{\prime }}+1}$. Correlation coefficients of ±1 were excluded due to undefined variance under the Fisher transformation.

The standard error was determined by implementing pooled Fisher’s z′ values, calculated as $\sqrt{1/\sum (n_{i}-3)}$, assuming a 95% confidence interval. A single interval was calculated from the pooled standard error, since confidence intervals were not pooled directly. The two-tailed *p*-values from the Pearson correlation coefficients were converted to Z-scores and combined across cohorts using Stouffer’s Z-method, assuming weight as *n* − 3 to preserve direction. Combined Z-scores were then converted into two-tailed *p*-values.

The within-group *p*-values were used to assess evidence for gene-expression associations within each *PROS1* expression condition since ΔR is a descriptive measure to characterize the magnitude and direction of changes in correlation, rather than as a formally tested endpoint. Similarly, multiplicity correction was not applied because the analysis was exploratory and intended to generate hypotheses for subsequent investigation, rather than to establish confirmatory gene-specific findings. We acknowledge that within-group *p*-values alone are not a confirmatory test of differential correlation. Accordingly, our interpretation of ΔR is restricted to descriptive changes in correlation patterns, and these differences only describe statistically significant differential correlations.

Weighting by (*n* − 3) was applied to the effect size and *p*-value pooling procedures to ensure that larger cohorts contributed proportionally more to pooled estimates. A heterogeneity assessment was performed using Cochran’s Q and *I*^2^ metric for each gene. Consequently, the pooled estimates and confidence intervals are determined by the weighted contributions of all included cohorts rather than by the arithmetic midpoint of the individual cohort estimates. Therefore, the pooled estimate need not coincide with the midpoint of the confidence interval, as the interval is calculated from the pooled estimate and its standard error. All analyses were performed in GraphPad Prism v.10 and Microsoft Excel. The above methodology is based on the pooling conducted by Prouse et al. [33].

### 4.5. Multi-Gene Analysis for Nsclc Adenocarcinoma, Ovarian Serous Cystadenocarcinoma, and Pdac

Cohorts that met each of the following criteria: low to moderate improvement with at least 1 hypoxia marker (R < −0.200); inverse relationship of expression with *PROS1* and genomic instability markers with at least 1 genomic instability marker (R <−0.200); and survival improvement with high *PROS1* expression. Since this paper aims to evaluate anti-tumor signaling similar to what has been previously explored in pancreatic ductal adenocarcinoma, these criteria had to be met to compare potential signaling similarities between cancers that meet these criteria. The two cancers that met these criteria were NSCLC adenocarcinoma and ovarian serous cystadenocarcinoma. The total gene list evaluated is listed in Supplementary Table S12.

In addition, the correlation coefficient was examined and interpreted to understand the relationship between variables. Correlation coefficients below 0.3 are regarded as indicating no significant or only a weak relationship [35,84,85]. Therefore, gene correlations falling below this threshold under both high and low *PROS1* conditions were viewed as having independent expression [84,85]. Direction of change was defined conservatively based on the difference between high and low *PROS1* expression states for each gene (∆*R* = *R_high_* − *R_low_*). The aforementioned is similar to Prouse et al. [33], however, the Pearson correlation coefficient was implemented since data in these cohorts were best interpreted under parametric conditions. Ultimately, this approach provides a descriptive measure of how the direction and magnitude of gene-expression associations differ between low and high-*PROS1* expression conditions.

Overall, this approach is derived from McKenzie et al., where differential correlation is a direct comparison of correlation coefficients between two distinct conditions and is also based on a biological approach to the interpretation of the correlation coefficient as described by Akoglu and Mukaka [33,84,85,86]. Therefore, only correlation coefficients that were ≥0.3 in magnitude in either low and/or high *PROS1* expression conditions can be interpreted appropriately with ΔR criteria. Thus, conservative associations were classified as follows in Table 10.

After the values were determined and interpreted accordingly, a heterogeneity analysis with Cochran’s Q statistic, Q *p*-value, and the *I*^2^ metric was performed for each gene. Genes with high heterogeneity in low-expression *PROS1* and/or high-expression *PROS1* strata were eliminated as significant, whether or not the magnitude of ΔR was significant. All statistically significant gene correlation data for NSCLC adenocarcinoma, ovarian serous cystadenocarcinoma, and PDAC cohorts are shown in Supplementary Tables S3, S4, and S5, respectively. The heterogeneity calculations for each gene of statistical significance evaluated in the NSCLC adenocarcinoma, ovarian serous cystadenocarcinoma, and PDAC cohorts are shown in Supplementary Tables S6, S7, and S8, respectively. The heterogeneity analyses for genes that were eliminated due to high heterogeneity for NSCLC lung adenocarcinoma, ovarian serous cystadenocarcinoma, and PDAC cohorts are shown in Tables S9, S10, and S11, respectively.

## 5. Conclusions

In summary, high intratumoral *PROS1* expression was associated with improved survival, lower hypoxia-signature marker scores, and reduced measures of genomic instability in PDAC, lung adenocarcinoma, and ovarian serous cystadenocarcinoma. Survival analyses were conducted via exploratory, unadjusted Mantel-Haenszel hazard ratio analyses, and the independent prognostic value of *PROS1* remains to be established in PDAC, lung adenocarcinoma, and ovarian serous cystadenocarcinoma. These cancers also exhibited *PROS1*-associated shifts in genes related to cell-cycle regulation, EMT, extracellular matrix remodeling, and hypoxia-associated metabolism. The data support a model in which high *PROS1* expression may lead to a less hypoxic and less genomically unstable tumor state in selected malignancies. This model is tumor-context dependent and remains hypothesis-generating. Mechanistic studies are now needed to determine whether PS directly modifies tumor perfusion, coagulation, hypoxia signaling, genome maintenance, EMT plasticity, or tumor progression in PDAC, lung adenocarcinoma, and ovarian serous cystadenocarcinoma.

## Figures and Tables

**Table 1:** Association of high v. low intratumoral *PROS1* expression with overall survival across cancer types. Pooled HRs are shown for high relative to low *PROS1* expression. Hazard ratios (HR) are shown as high relative to low *PROS1* expression. Hazard ratios < 1 indicate improved survival in the high-*PROS1* group, whereas hazard ratios > 1 indicate poorer survival in the high-PROS1 group. PDAC results have been published previously and are not included in the table.

Cancer Type	Pooled Hazard Ratio (High/Low)	Pooled 95% CI	Pooled *p*-Value
**Bladder urothelial carcinoma**	1.637	1.558–1.717	4.8 × 10^−9^
**Cervical squamous cell carcinoma**	1.869	1.642–2.095	3.5 × 10^−3^
**Esophageal adenocarcinoma**	1.878	1.675–2.080	1.8 × 10^−3^
**NSCLC adenocarcinoma**	0.679	0.553–0.805	2.2 × 10^−3^
**NSCLC squamous cell carcinoma**	1.420	1.299–1.540	3.2 × 10^−3^
**Ovarian serous cystadenocarcinoma**	0.681	0.557–0.804	1.5 × 10^−3^
**Renal clear cell carcinoma**	0.675	0.553–0.797	9.2 × 10^−4^
**Renal non-clear cell carcinoma**	2.027	1.778–2.275	4.4 × 10^−3^
**Stomach adenocarcinoma**	1.577	1.424–1.730	1.3 × 10^−3^

**Table 2:** Pooled Pearson correlation coefficients between *PROS1* expression and tumor mutational burden (TMB). Negative coefficients indicate lower TMB in tumors with higher *PROS1* expression. All data have been pooled between cohorts. Note that ** indicates the ovarian serous cystadenocarcinoma TMB value was not pooled due to insufficient TMB data in the TCGA GDC cohort.

Cancer Type	Tumor Mutational Burden (TMB)	Pooled 95% CI	Pooled *p*-Value
**Bladder urothelial carcinoma**	−0.123	−0.173 to −0.072	2.8 × 10^−6^
**Cervical squamous cell carcinoma**	−0.133	−0.221 to −0.041	4.2 × 10^−3^
**Esophageal adenocarcinoma**	−0.155	−0.260 to −0.045	5.4 × 10^−3^
**NSCLC adenocarcinoma**	−0.231	−0.302 to −0.150	1.6 × 10^−6^
**NSCLC squamous cell carcinoma**	−0.115	−0.176 to −0.052	8.0 × 10^−4^
** Ovarian serous cystadenocarcinoma	−0.204	−0.326 to −0.0746	2.2 × 10^−3^
**Pancreatic adenocarcinoma**	−0.429	−0.474 to −0.329	1.0 × 10^−4^
**Renal clear cell carcinoma**	0.002	−0.071 to 0.076	3.5 × 10^−3^
**Renal non-clear cell carcinoma**	−0.011	−0.097 to 0.079	4.0 × 10^−4^
**Stomach adenocarcinoma**	−0.181	−0.246 to −0.111	3.9 × 10^−6^

**Table 3:** Pearson correlation coefficients between intratumoral *PROS1* expression and tumor break-load (TBL) score. Note that * indicates estimates pooled across two or more cohorts. TBL score data were unavailable for cervical squamous cell carcinoma, esophageal adenocarcinoma, and NSCLC adenocarcinoma.

Cancer Type	Tumor Break-Load (TBL) Score	95% CI	*p*-Value
**Bladder urothelial carcinoma**	−0.187	−0.283 to −0.089	3.0 × 10^−4^
**NSCLC squamous cell carcinoma**	−0.142	−0.231 to −0.050	2.5 × 10^−3^
**Ovarian serous cystadenocarcinoma**	−0.208	−0.317 to −0.093	5.0 × 10^−4^
* Pancreatic adenocarcinoma	−0.622	−0.634 to −0.459	1.0 × 10^−4^
**Renal clear cell carcinoma**	0.098	0.006 to 0.189	3.6 × 10^−3^
**Renal non-clear cell carcinoma**	0.1341	0.012 to 0.253	3.2 × 10^−2^
**Stomach adenocarcinoma**	0.1215	0.025 to 0.216	1.4 × 10^−2^

**Table 4:** Pearson correlation coefficients between intratumoral *PROS1* expression and aneuploidy score (AS). Note that * indicates estimates pooled across two or more cohorts.

Cancer Type	Aneuploidy Score (AS)	95% CI	*p*-Value
* Bladder urothelial carcinoma	−0.126	−0.202 to −0.047	1.8 × 10^−3^
**Cervical squamous cell carcinoma**	−0.145	−0.262 to −0.024	4.9 × 10^−2^
**Esophageal adenocarcinoma**	−0.168	−0.317 to −0.011	3.5 × 10^−2^
**NSCLC adenocarcinoma**	−0.241	−0.329 to −0.149	5.8 × 10^−3^
**NSCLC squamous cell carcinoma**	−0.119	−0.209 to −0.027	1.2 × 10^−2^
**Ovarian serous cystadenocarcinoma**	−0.149	−0.265 to −0.028	1.6 × 10^−2^
* Pancreatic adenocarcinoma	−0.581	−0.621 to −0.410	1.0 × 10^−4^
**Renal clear cell carcinoma**	0.147	0.058 to 0.233	1.2 × 10^−3^
**Renal non-clear cell carcinoma**	0.120	0.003 to 0.234	4.4 × 10^−2^
**Stomach adenocarcinoma**	−0.113	−0.212 to −0.010	3.1 × 10^−2^

**Table 5:** Pearson correlation coefficients between *PROS1* expression and hypoxia-signature scores. Note that * indicates estimates pooled across two or more cohorts. Esophageal adenocarcinoma and stomach adenocarcinoma were not included because hypoxia-signature data were unavailable.

Cancer	Buffa Hypoxia Score		Winter Hypoxia Score		Ragnum Hypoxia Score	
	R-Value	*p*-Value	R-Value	*p*-Value	R-Value	*p*-Value
* Bladder urothelial carcinoma	−0.114	4.0 × 10^−4^	−0.127	1.7 × 10^−3^	−0.139	4.1 × 10^−4^
**Cervical squamous cell carcinoma**	−0.120	4.9 × 10^−2^	−0.135	3.0 × 10^−2^	0.124	4.0 × 10^−2^
**NSCLC adenocarcinoma**	−0.318	1.0 × 10^−4^	−0.304	1.0 × 10^−4^	−0.316	1.0 × 10^−4^
**NSCLC squamous cell carcinoma**	−0.250	1.0 × 10^−4^	−0.151	1.3 × 10^−3^	−0.260	1.0 × 10^−4^
**Ovarian serous cystadenocarcinoma**	−0.311	1.0 × 10^−4^	−0.212	4.0 × 10^−4^	−0.253	1.0 × 10^−4^
**Pancreatic adenocarcinoma**	−0.302	1.0 × 10^−4^	−0.310	1.0 × 10^−4^	−0.301	1.0 × 10^−4^
**Renal clear cell carcinoma**	0.256	1.0 × 10^−3^	0.144	1.0 × 10^−3^	0.149	7.0 × 10^−4^
**Renal non-clear cell carcinoma**	−0.128	3.6 × 10^−2^	−0.126	4.1 × 10^−2^	−0.126	3.8 × 10^−2^

**Table 6:** Pooled ΔR values for high- and low-*PROS1* expression conditions, pooled two-tailed *p*-values, significance, and biological interpretation for genes in NSCLC lung adenocarcinoma human RNA-sequencing data. Genes that demonstrate a statistically significant correlation with a biologically significant change in R magnitude under conditions of *PROS1* expression are shown.

Gene	Low *PROS1* R-Value	High *PROS1* R-Value	ΔR	Low *PROS1* *p*-Value	High *PROS1* *p*-Value	Biological Interpretation
***MMP2***	0.328	−0.200	−0.528	4.7 × 10^−10^	6.4 × 10^−4^	Moderate magnitude ΔR
***ANAPC2***	0.284	−0.330	−0.613	3.5 × 10^−9^	1.0 × 10^−6^	High magnitude ΔR
***SNAI2***	0.324	−0.311	−0.635	2.2 × 10^−10^	5.3 × 10^−7^	High magnitude ΔR
***MMP9***	0.338	−0.335	−0.672	1.7 × 10^−10^	3.0 × 10^−7^	High magnitude ΔR

**Table 7:** Pooled ΔR values for high- and low-*PROS1* expression conditions, pooled two-tailed *p*-values, significance, and biological interpretation for genes in ovarian serous cystadenocarcinoma human RNA-sequencing data. Genes that demonstrate a statistically significant correlation with a biologically significant change in R magnitude under conditions of *PROS1* expression are shown.

Gene	Low *PROS1* R-Value	High *PROS1* R-Value	ΔR	Low *PROS1* *p*-Value	High *PROS1* *p*-Value	Biological Interpretation
***SNAI1***	0.320	−0.187	−0.507	6.3 × 10^−6^	7.5 × 10^−3^	Moderate magnitude ΔR
***MMP7***	0.343	−0.185	−0.529	6.7 × 10^−7^	7.4 × 10^−3^	Moderate magnitude ΔR
***ANAPC2***	0.360	−0.199	−0.559	3.4 × 10^−6^	3.1 × 10^−3^	Moderate magnitude ΔR
***SLC2A1***	0.407	−0.199	−0.606	1.5 × 10^−6^	2.2 × 10^−3^	High magnitude ΔR
***MMP9***	0.319	−0.295	−0.614	4.2 × 10^−6^	7.4 × 10^−5^	High magnitude ΔR
***SNAI2***	0.381	−0.302	−0.683	5.5 × 10^−7^	1.4 × 10^−5^	High magnitude ΔR

**Table 8:** Pooled ΔR values for high- and low-*PROS1* expression conditions, pooled two-tailed *p*-values, significance, and biological interpretation for genes in pancreatic ductal adenocarcinoma human RNA-sequencing data. Genes that demonstrate a statistically significant correlation with a biologically significant change in R magnitude under conditions of *PROS1* expression are shown.

Gene	Low *PROS1* R-Value	High *PROS1* R-Value	ΔR	Low *PROS1* *p*-Value	High *PROS1* *p*-Value	Biological Interpretation
***KNL1***	0.224	−0.253	−0.477	2.7 × 10^−2^	6.7 × 10^−2^	Moderate magnitude ΔR
***AURKC***	0.169	−0.328	−0.498	6.3 × 10^−5^	3.4 × 10^−6^	Moderate magnitude ΔR
***ANAPC2***	0.302	−0.291	−0.593	6.7 × 10^−4^	2.2 × 10^−5^	Moderate magnitude ΔR
***CAMSAP3***	0.314	−0.328	−0.641	1.0 × 10^−5^	2.5 × 10^−6^	High magnitude ΔR
***ANAPC4***	0.428	−0.278	−0.704	4.2 × 10^−10^	1.9 × 10^−3^	High magnitude ΔR

**Table 9:** List of cohorts by cancer type, name, and total number of patients.

Cancer Type	Cohort Name	Patient Number
**Bladder urothelial carcinoma**	TCGA, PanCancer Atlas	407
	MSK/TCGA Euro Urol 2020	294
	TCGA Cell 2017	404
	TCGA GDC, 2025	401
**Cervical squamous cell carcinoma**	TCGA, PanCancer Atlas	276
	TCGA GDC, 2025	307
**Esophageal adenocarcinoma**	TCGA, PanCancer Atlas	183
	TCGA GDC, 2025	157
**NSCLC adenocarcinoma**	TCGA, PanCancer Atlas	441
	CPTAC GDC 2025	192
	TCGA GDC, 2025	478
**NSCLC squamous cell carcinoma**	TCGA, PanCancer Atlas	454
	CPTAC GDC 2025	95
	TCGA GDC, 2025	464
**Ovarian serous cystadenocarcinoma**	TCGA, PanCancer Atlas	216
	TCGA GDC 2025	418
**Pancreatic adenocarcinoma**	CPTAC, Cell 2021	140
	CPTAC GDC, 2025	161
	TCGA, PanCancer Atlas	178
	TCGA GDC, 2025	179
**Renal clear cell carcinoma**	TCGA, PanCancer Atlas	495
	TCGA GDC, 2025	347
**Renal non-clear cell carcinoma**	TCGA, PanCancer Atlas	281
	TCGA GDC, 2025	243
**Stomach adenocarcinoma**	TCGA, PanCancer Atlas	410
	TCGA GDC, 2025	382

Note that CPTAC GDC, 2025 was excluded from survival data analysis due to insufficient survival data, and data from this cohort were used to generate a Pearson correlation coefficient associated with TMB.

**Table 10:** Classification of direction of change and conservative biological associations.

Association	Criteria	Biological Interpretation
**Independent correlation**	|∆*R*| < 0.20	*PROS1*-independent correlation
**Strengthened**	|∆*R*| ≥ 0.30	Stronger positive gene-expression correlation in high-*PROS1* expressing tumors
**Lower magnitude ΔR**	0.30 < |∆*R*| < 0.40	High *PROS1* tumors exhibit weaker gene-expression correlations
**Moderate magnitude ΔR**	0.40 ≤ |∆*R*| < 0.59	High *PROS1* tumors exhibit moderate gene-expression correlations
**High magnitude ΔR**	0.60 ≤ |∆*R*| < 1.00	High *PROS1* tumors exhibit strong gene-expression correlations

## Data Availability

All data supporting reported results can be found on cBioPortal https://www.cbioportal.org (accessed on 1 June 2026).

## Supplementary Materials

The following supporting information can be downloaded at: https://www.mdpi.com/article/10.3390/ijms27219613/s1.
