# Supplementary material for: High Intratumoral PROS1 Expression Is Associated with Improved Survival, Reduced Hypoxia Score Markers, and Alterations in Gene Expression Associated with Oncogene Signaling and Cell Cycle Signaling in Selected Solid Tumors
Source: Int J Mol Sci. 2026 Oct 27;27(21):9613. doi: 10.3390/ijms27219613 (PMC13762795; doi:10.3390/ijms27219613)
Supplement: Supplementary file 1 [file ijms-27-09613-s001.zip › ijms-4621256-supplementary.pdf]

Supplementary Data

Supplementary Table S1. Results from Cox proportional hazards regression for each cancer type, excluding pancreatic adenocarcinoma (previously published).

Cancer Type	Cohort	exp(B1)	95% CI
**Bladder urothelial carcinoma**	TCGA Cell 2017	0.9852	0.9764 to 0.9928
	TCGA GDC, 2025	0.9862	0.9775 to 0.9937
	MSK/TCGA Euro Urol 20202020	1.006	1.002 to 1.009
	TCGA, PanCancer Atlas	0.9862	0.9777 to 0.9936
**Cervical squamous cell carcinoma**	TCGA, PanCancer Atlas	0.9967	0.9876 to 1.004
	TCGA GDC, 2025	0.996	0.9874 to 1.003
**Esophageal adenocarcinoma**	TCGA, PanCancer Atlas	0.9945	0.9784 to 1.008
	TCGA GDC, 2025	0.9886	0.9704 to 1.003
**NSCLC adenocarcinoma**	TCGA, PanCancer Atlas	0.9934	0.9863 to 0.9998
	TCGA GDC, 2025	1.000	0.9969 to 1.003
**NSCLC squamous cell carcinoma**	TCGA, PanCancer Atlas	0.9976	0.9922 to 1.003
	TCGA GDC, 2025	0.9946	0.9892 to 0.9995
**Ovarian serous cystadenocarcinoma**	TCGA, PanCancer Atlas	1.001	0.9958 to 1.006
	TCGA GDC, 2025	1.001	0.9956 to 1.006
**Renal clear cell carcinoma**	TCGA, PanCancer Atlas	1.004	1.000 to 1.007
	TCGA GDC, 2025	1.004	1.000 to 1.007
**Renal non-clear cell carcinoma**	TCGA, PanCancer Atlas	0.9957	0.9836 to 1.006
	TCGA GDC, 2025	0.9978	0.9870 to 1.007
**Stomach adenocarcinoma**	TCGA, PanCancer Atlas	0.9788	0.9645 to 0.9917
	TCGA GDC, 2025	0.9746	0.9595 to 0.9885

Supplementary Table S2. Pooled Pearson correlation coefficients between *PROS1* expression and microsatellite instability MANTIS score. All data have been pooled between cohorts. Note that * represents pooled values.

Cancer Type	Microsatellite Instability Score (MSI)	Pooled 95% CI	Pooled p-value
*Bladder urothelial carcinoma	0.012	-0.071 to 0.095	3.2×10^{-2}
Cervical squamous cell carcinoma	-0.046	-0.159 to 0.069	4.4×10^{-1}
Esophageal adenocarcinoma	-0.053	-0.260 to -0.045	4.8×10^{-1}
NSCLC adenocarcinoma	-0.089	-0.181 to 0.004	6.3×10^{-2}
NSCLC squamous cell carcinoma	-0.023	-0.112 to 0.068	6.1×10^{-1}
Ovarian serous cystadenocarcinoma	-0.048	-0.180 to -0.086	4.9×10^{-1}
Pancreatic adenocarcinoma	0.013	-0.094 to 0.119	7.0×10^{-1}
Renal clear cell carcinoma	0.005	-0.104 to 0.114	9.3×10^{-1}
Renal non-clear cell carcinoma	0.030	-0.088 to 0.146	6.2×10^{-1}
Stomach adenocarcinoma	-0.180	-0.272 to -0.085	2.0×10^{-4}

Supplementary Table S3. Pooled delta-r values for high- and low-*PROS1* expression conditions, pooled two-tailed p-values, significance, and biological interpretation for genes in NSCLC lung adenocarcinoma human RNA-sequencing data. Genes that demonstrate a statistically significant correlation independent of *PROS1* expression are shown.

Gene Name	Low *PROS1* pooled R-value	High *PROS1* pooled R-value	ΔR	Low *PROS1* pooled p-value	High *PROS1* pooled p-value	Biological Interpretation
AURKB	-0.271	0.157	0.428	1.4×10^{-10}	7.6×10^{-3}	Independent Correlation
GAS2L3	-0.211	0.217	0.427	6.2×10^{-7}	6.1×10^{-5}	Independent Correlation
MAD2L1	-0.191	0.207	0.398	3.7×10^{-6}	3.4×10^{-4}	Independent Correlation
KIF18A	-0.170	0.222	0.393	3.1×10^{-5}	4.2×10^{-5}	Independent Correlation

NRAS	-0.178	0.213	0.391	2.3×10^{-5}	1.1×10^{-4}	Independent Correlation
SMC2	-0.204	0.183	0.387	5.2×10^{-7}	9.8×10^{-4}	Independent Correlation
STAG1	-0.178	0.202	0.380	4.3×10^{-5}	1.8×10^{-4}	Independent Correlation
BUB3	-0.177	0.198	0.375	2.1×10^{-5}	4.0×10^{-4}	Independent Correlation
RAD21	-0.159	0.208	0.367	1.0×10^{-4}	4.4×10^{-4}	Independent Correlation
MYC	-0.193	0.166	0.359	7.0×10^{-4}	3.2×10^{-3}	Independent Correlation
PFKFB4	-0.149	0.204	0.353	9.2×10^{-4}	2.4×10^{-4}	Independent Correlation
HK2	-0.147	0.203	0.350	8.2×10^{-4}	2.8×10^{-4}	Independent Correlation
KRAS	-0.181	0.165	0.346	2.6×10^{-3}	4.8×10^{-3}	Independent Correlation
MAD2L1BP	-0.163	0.170	0.333	4.5×10^{-3}	6.7×10^{-5}	Independent Correlation
SMC3	-0.148	0.179	0.327	4.3×10^{-4}	1.6×10^{-3}	Independent Correlation
SMC1A	-0.157	0.166	0.324	1.8×10^{-4}	2.3×10^{-3}	Independent Correlation
SPC24	-0.190	0.075	0.265	2.6×10^{-6}	6.6×10^{-6}	Independent Correlation
INCENP	-0.222	-0.151	0.071	2.2×10^{-7}	8.1×10^{-4}	Independent Correlation
BUB1B	-0.217	-0.149	0.068	2.2×10^{-7}	9.9×10^{-4}	Independent Correlation
NCAPD2	-0.243	-0.178	0.065	1.6×10^{-8}	8.5×10^{-5}	Independent Correlation
CCNB2	-0.233	-0.170	0.063	2.0×10^{-8}	1.5×10^{-4}	Independent Correlation
CKAP5	-0.205	-0.147	0.058	1.2×10^{-6}	1.2×10^{-3}	Independent Correlation
ANAPC7	-0.263	-0.205	0.058	2.6×10^{-9}	1.4×10^{-6}	Independent Correlation
CDC20	-0.214	-0.157	0.056	2.5×10^{-7}	4.6×10^{-4}	Independent Correlation
NUF2	-0.252	-0.196	0.056	1.0×10^{-9}	6.1×10^{-4}	Independent Correlation
CDK1	-0.235	-0.181	0.054	5.8×10^{-9}	4.5×10^{-5}	Independent Correlation
TTK	-0.212	-0.159	0.053	2.8×10^{-7}	4.2×10^{-4}	Independent Correlation
KIF15	-0.212	-0.159	0.053	2.6×10^{-7}	4.0×10^{-4}	Independent Correlation
NDC80	-0.180	-0.128	0.052	9.6×10^{-6}	5.7×10^{-5}	Independent Correlation
SLC2A1	-0.195	-0.146	0.049	5.9×10^{-4}	1.3×10^{-3}	Independent Correlation
KIF23	-0.217	-0.171	0.045	1.5×10^{-7}	4.5×10^{-5}	Independent Correlation
NCAPH	-0.215	-0.171	0.045	1.5×10^{-7}	1.5×10^{-4}	Independent Correlation
CENPA	-0.261	-0.218	0.043	2.3×10^{-9}	6.7×10^{-6}	Independent Correlation
CDCA8	-0.197	-0.156	0.041	1.7×10^{-6}	6.0×10^{-4}	Independent Correlation

NCAPG	-0.199	-0.160	0.039	1.1×10^{-6}	3.7×10^{-4}	Independent Correlation
BIRC5	-0.252	-0.213	0.038	5.4×10^{-10}	1.4×10^{-6}	Independent Correlation
KIF11	-0.203	-0.167	0.036	6.4×10^{-7}	1.9×10^{-4}	Independent Correlation
CCNB1	-0.237	-0.204	0.034	6.6×10^{-9}	5.9×10^{-6}	Independent Correlation
E2F1	-0.224	-0.191	0.033	3.5×10^{-8}	5.1×10^{-6}	Independent Correlation
RCC1	-0.194	-0.161	0.032	2.0×10^{-6}	3.4×10^{-4}	Independent Correlation
CDC25C	-0.176	-0.145	0.031	2.0×10^{-5}	1.7×10^{-3}	Independent Correlation
NCAPD3	-0.191	-0.161	0.030	4.5×10^{-6}	2.1×10^{-4}	Independent Correlation
DCTN1	0.185	0.214	0.028	1.8×10^{-5}	1.0×10^{-4}	Independent Correlation
TPX2	-0.218	-0.191	0.027	7.6×10^{-8}	2.4×10^{-5}	Independent Correlation
CLASP1	-0.168	-0.141	0.027	6.4×10^{-5}	2.1×10^{-3}	Independent Correlation
CENPB	-0.154	-0.129	0.025	3.0×10^{-4}	7.6×10^{-5}	Independent Correlation
CENPE	-0.187	-0.163	0.024	4.2×10^{-6}	9.4×10^{-5}	Independent Correlation
KIF4A	-0.201	-0.179	0.021	9.9×10^{-7}	8.1×10^{-5}	Independent Correlation
CENPF	-0.211	-0.191	0.020	4.4×10^{-7}	5.2×10^{-6}	Independent Correlation
SPC25	-0.209	-0.190	0.019	2.8×10^{-7}	2.8×10^{-5}	Independent Correlation
MKI67	-0.183	-0.164	0.019	1.0×10^{-5}	2.8×10^{-4}	Independent Correlation
ZWINT	-0.222	-0.204	0.018	7.0×10^{-8}	7.8×10^{-6}	Independent Correlation
SMC4	-0.190	-0.177	0.013	5.4×10^{-6}	1.1×10^{-3}	Independent Correlation
IL6	0.153	0.166	0.013	7.7×10^{-4}	2.9×10^{-4}	Independent Correlation
PDK1	-0.145	-0.133	0.012	5.3×10^{-4}	3.0×10^{-3}	Independent Correlation
PLK1	-0.194	-0.186	0.008	2.0×10^{-6}	4.5×10^{-5}	Independent Correlation
TACC3	-0.190	-0.184	0.006	3.8×10^{-6}	4.9×10^{-5}	Independent Correlation
PLK4	-0.167	-0.162	0.005	5.2×10^{-5}	3.8×10^{-4}	Independent Correlation
KNL1	-0.191	-0.189	0.002	4.5×10^{-6}	7.4×10^{-6}	Independent Correlation
PTK2	-0.153	-0.153	0.001	5.3×10^{-4}	8.5×10^{-3}	Independent Correlation
DYNC1H1	-0.162	-0.162	0.000	1.9×10^{-4}	3.9×10^{-3}	Independent Correlation
ZWILCH	-0.170	-0.170	0.000	3.8×10^{-5}	2.0×10^{-4}	Independent Correlation
PLK2	0.195	0.192	-0.003	3.5×10^{-6}	2.9×10^{-6}	Independent Correlation
ANAPC10	0.171	0.164	-0.007	2.2×10^{-3}	1.2×10^{-4}	Independent Correlation

AKT1	-0.147	-0.154	-0.007	6.0×10^{-4}	7.3×10^{-4}	Independent Correlation
EPAS1	0.166	0.158	-0.008	5.9×10^{-5}	4.6×10^{-4}	Independent Correlation
AURKA	-0.220	-0.229	-0.009	9.0×10^{-8}	5.4×10^{-6}	Independent Correlation
CENPC	0.187	0.176	-0.011	4.4×10^{-6}	2.8×10^{-5}	Independent Correlation
MIS12	-0.141	-0.153	-0.012	8.4×10^{-4}	3.9×10^{-4}	Independent Correlation
PROM1	0.178	0.164	-0.013	4.2×10^{-5}	1.5×10^{-4}	Independent Correlation
MAPRE1	-0.148	-0.163	-0.015	1.1×10^{-3}	3.8×10^{-4}	Independent Correlation
TYRO3	0.246	0.229	-0.017	6.9×10^{-6}	1.0×10^{-7}	Independent Correlation
WAPL	0.211	0.179	-0.032	1.7×10^{-6}	1.6×10^{-5}	Independent Correlation
NUMA1	-0.175	-0.214	-0.039	2.9×10^{-3}	1.3×10^{-4}	Independent Correlation
TNF	0.272	0.191	-0.080	1.3×10^{-9}	4.2×10^{-5}	Independent Correlation
ESCO1	0.190	0.100	-0.089	1.2×10^{-3}	4.1×10^{-3}	Independent Correlation
CAMSAP3	-0.153	-0.251	-0.098	3.6×10^{-4}	6.7×10^{-6}	Independent Correlation
SNAI1	0.212	0.067	-0.145	2.1×10^{-4}	1.1×10^{-3}	Independent Correlation
TWIST1	0.167	-0.141	-0.308	4.3×10^{-3}	2.5×10^{-3}	Independent Correlation
SRC	0.172	-0.173	-0.345	6.0×10^{-5}	3.4×10^{-3}	Independent Correlation
CDH2	0.174	-0.189	-0.363	2.3×10^{-4}	9.2×10^{-4}	Independent Correlation
PFKFB3	0.195	-0.187	-0.383	4.5×10^{-6}	5.4×10^{-4}	Independent Correlation
CDH1	0.184	-0.206	-0.389	1.4×10^{-5}	1.8×10^{-4}	Independent Correlation
CD44	0.213	-0.177	-0.390	2.5×10^{-7}	3.2×10^{-3}	Independent Correlation
HIF1A	0.200	-0.236	-0.436	3.2×10^{-6}	7.5×10^{-5}	Independent Correlation
VEGFA	0.203	-0.254	-0.457	7.6×10^{-6}	1.9×10^{-5}	Independent Correlation
TGFB1	0.273	-0.208	-0.480	6.0×10^{-10}	5.5×10^{-4}	Independent Correlation
CA9	0.236	-0.251	-0.487	2.1×10^{-4}	1.1×10^{-7}	Independent Correlation

Supplementary Table S4. Pooled delta-r values for high- and low-*PROS1* expression conditions, pooled two-tailed p-values, significance, and biological interpretation for genes in ovarian serous cystadenocarcinoma human RNA-sequencing data. Genes that demonstrate a statistically significant correlation independent of *PROS1* expression are shown.

Gene Name	Low *PROS1* pooled R-value	High *PROS1* pooled R-value	ΔR	Low *PROS1* pooled p-value	High *PROS1* pooled p-value	Biological Interpretation
PROM1	-0.057	0.238	0.295	3.1×10^{-3}	4.6×10^{-4}	Independent Correlation
VIM	0.161	0.310	0.149	6.7×10^{-3}	7.9×10^{-6}	Independent Correlation
CASP3	0.225	0.276	0.051	1.7×10^{-4}	1.2×10^{-5}	Independent Correlation
PLK2	0.210	0.254	0.044	2.8×10^{-4}	2.5×10^{-5}	Independent Correlation
ANAPC5	0.220	0.229	0.009	1.6×10^{-4}	1.6×10^{-4}	Independent Correlation
CDH2	0.183	0.177	-0.005	2.2×10^{-3}	5.1×10^{-3}	Independent Correlation
CAMSAP3	0.220	0.215	-0.005	2.0×10^{-4}	2.5×10^{-4}	Independent Correlation
HK2	0.194	0.187	-0.006	2.1×10^{-3}	1.3×10^{-3}	Independent Correlation
KIF15	0.230	0.219	-0.011	1.2×10^{-4}	2.4×10^{-4}	Independent Correlation
ANAPC10	0.198	0.186	-0.012	2.5×10^{-3}	2.5×10^{-3}	Independent Correlation
CENPE	0.222	0.202	-0.020	1.6×10^{-4}	1.1×10^{-3}	Independent Correlation
ANAPC1	0.276	0.237	-0.038	7.3×10^{-6}	5.4×10^{-5}	Independent Correlation
ANAPC4	0.230	0.188	-0.041	1.4×10^{-4}	1.6×10^{-3}	Independent Correlation
TNF	0.250	0.207	-0.043	1.8×10^{-4}	3.6×10^{-4}	Independent Correlation
PFKFB4	0.218	0.161	-0.056	4.1×10^{-4}	7.0×10^{-3}	Independent Correlation
CENPF	0.244	0.182	-0.062	3.8×10^{-5}	5.8×10^{-3}	Independent Correlation
TWIST1	0.268	0.195	-0.073	2.5×10^{-5}	1.8×10^{-3}	Independent Correlation
NRAS	0.311	0.168	-0.143	4.6×10^{-6}	5.4×10^{-3}	Independent Correlation
CD4	0.042	-0.178	-0.220	5.3×10^{-3}	3.7×10^{-3}	Independent Correlation
SPC25	0.234	-0.044	-0.278	1.3×10^{-4}	1.5×10^{-5}	Independent Correlation
AURKC	0.165	-0.180	-0.345	6.5×10^{-3}	8.1×10^{-3}	Independent Correlation
CDCA8	0.162	-0.184	-0.346	6.9×10^{-3}	2.8×10^{-3}	Independent Correlation
KIFC1	0.173	-0.174	-0.348	5.1×10^{-3}	6.2×10^{-3}	Independent Correlation
NCAPH	0.191	-0.162	-0.354	1.3×10^{-3}	8.9×10^{-3}	Independent Correlation
NUF2	0.189	-0.176	-0.365	2.2×10^{-3}	4.9×10^{-3}	Independent Correlation
CENPB	0.195	-0.170	-0.365	1.2×10^{-3}	5.5×10^{-3}	Independent Correlation
MAD2L1BP	0.175	-0.194	-0.369	4.8×10^{-3}	1.9×10^{-3}	Independent Correlation

TACC3	0.184	-0.190	-0.374	3.3×10^{-3}	1.8×10^{-3}	Independent Correlation
CDC20	0.181	-0.199	-0.380	2.1×10^{-3}	1.1×10^{-3}	Independent Correlation
MKI67	0.178	-0.203	-0.381	3.5×10^{-3}	7.7×10^{-4}	Independent Correlation
CDK1	0.189	-0.195	-0.384	1.4×10^{-3}	1.6×10^{-3}	Independent Correlation
KIF11	0.218	-0.167	-0.385	1.6×10^{-4}	8.7×10^{-3}	Independent Correlation
CKAP5	0.211	-0.177	-0.389	4.2×10^{-4}	4.6×10^{-3}	Independent Correlation
NCAPD2	0.169	-0.230	-0.399	5.7×10^{-3}	8.0×10^{-5}	Independent Correlation
PLK1	0.212	-0.188	-0.400	3.5×10^{-4}	2.5×10^{-3}	Independent Correlation
NOTCH1	0.197	-0.204	-0.400	1.4×10^{-3}	2.0×10^{-3}	Independent Correlation
INCENP	0.213	-0.188	-0.402	2.3×10^{-4}	1.5×10^{-3}	Independent Correlation
NDC80	0.231	-0.192	-0.423	1.3×10^{-4}	1.7×10^{-3}	Independent Correlation
BUB3	0.223	-0.205	-0.428	2.4×10^{-4}	1.3×10^{-3}	Independent Correlation
KNL1	0.185	-0.243	-0.429	2.3×10^{-3}	7.8×10^{-5}	Independent Correlation
IL1B	0.226	-0.203	-0.429	4.7×10^{-4}	1.3×10^{-3}	Independent Correlation
GAS2L3	0.242	-0.189	-0.431	1.4×10^{-4}	2.9×10^{-3}	Independent Correlation
KIF23	0.244	-0.188	-0.432	2.6×10^{-5}	2.1×10^{-3}	Independent Correlation
AURKA	0.234	-0.201	-0.436	9.5×10^{-5}	1.1×10^{-3}	Independent Correlation
CCNB1	0.210	-0.229	-0.438	4.3×10^{-4}	2.0×10^{-4}	Independent Correlation
SRC	0.242	-0.200	-0.442	9.3×10^{-5}	2.0×10^{-3}	Independent Correlation
MIS12	0.277	-0.173	-0.450	5.4×10^{-6}	6.9×10^{-3}	Independent Correlation
KIF2C	0.267	-0.183	-0.450	5.6×10^{-6}	3.0×10^{-3}	Independent Correlation
SPC24	0.223	-0.229	-0.452	1.9×10^{-4}	2.9×10^{-4}	Independent Correlation
STAT3	0.278	-0.189	-0.468	7.8×10^{-6}	4.0×10^{-3}	Independent Correlation
NCAPD3	0.197	-0.274	-0.471	9.5×10^{-4}	8.2×10^{-6}	Independent Correlation
KIF4A	0.256	-0.216	-0.472	1.2×10^{-5}	4.7×10^{-4}	Independent Correlation
CD8A	0.266	-0.207	-0.473	2.5×10^{-5}	5.7×10^{-4}	Independent Correlation
ZWINT	0.228	-0.246	-0.473	2.7×10^{-4}	6.0×10^{-4}	Independent Correlation
WAPL	0.289	-0.187	-0.476	3.6×10^{-6}	3.2×10^{-3}	Independent Correlation
ZWILCH	0.239	-0.243	-0.481	1.1×10^{-4}	4.0×10^{-5}	Independent Correlation
CA9	0.250	-0.251	-0.501	1.5×10^{-4}	6.7×10^{-4}	Independent Correlation

MERTK	0.293	-0.224	-0.517	1.9×10^{-6}	1.9×10^{-4}	Independent Correlation
HIF3A	0.294	-0.226	-0.520	5.8×10^{-6}	4.9×10^{-4}	Independent Correlation
BUB1B	0.290	-0.237	-0.528	1.6×10^{-6}	9.3×10^{-5}	Independent Correlation

Supplementary Table S5. Pooled delta-r values for high- and low-*PROS1* expression conditions, pooled two-tailed p-values, significance, and biological interpretation for genes in pancreatic ductal adenocarcinoma human RNA-sequencing data. Genes that demonstrate a statistically significant correlation independent of *PROS1* expression are shown.

Gene	Low *PROS1* R-value	High *PROS1* R-value	ΔR	Low *PROS1* p-value	High *PROS1* p-value	Biological Interpretation
RAD21	0.0313	0.2563	0.2250	1.7×10^{-5}	2.2×10^{-3}	Independent Correlation
NCAPD2	-0.4947	-0.3118	0.1829	8.8×10^{-11}	5.2×10^{-6}	Independent Correlation
CLASP2	0.2286	0.3551	0.1266	7.3×10^{-8}	6.2×10^{-8}	Independent Correlation
PDS5B	0.2317	0.3176	0.0859	2.4×10^{-8}	1.2×10^{-6}	Independent Correlation
SMC1A	0.3240	0.3984	0.0744	5.1×10^{-7}	9.6×10^{-9}	Independent Correlation
ESCO1	0.2417	0.3097	0.0680	4.7×10^{-2}	4.0×10^{-6}	Independent Correlation
MAPRE1	0.3458	0.3990	0.0532	6.4×10^{-8}	4.1×10^{-9}	Independent Correlation
ANAPC10	0.2279	0.2543	0.0263	5.9×10^{-7}	4.1×10^{-3}	Independent Correlation
ZWILCH	0.2394	0.2657	0.0263	8.0×10^{-4}	2.8×10^{-3}	Independent Correlation
WAPL	0.3816	0.3791	-0.0025	2.2×10^{-10}	3.3×10^{-8}	Independent Correlation
CLASP1	0.3318	0.3203	-0.0115	1.0×10^{-7}	4.4×10^{-6}	Independent Correlation
CENPA	-0.3432	-0.3572	-0.0140	5.1×10^{-7}	3.9×10^{-7}	Independent Correlation
GAS2L3	0.3278	0.3074	-0.0204	1.0×10^{-7}	5.6×10^{-6}	Independent Correlation
TOP2B	0.4725	0.3423	-0.1302	5.9×10^{-10}	1.1×10^{-7}	Independent Correlation

Supplementary Table S6. Heterogeneity statistics, including Cochran's Q-value, Cochran's Q p-value, and I^2 value as a percentage, for all genes in NSCLC lung adenocarcinoma human RNA-sequencing data that showed a statistically significant correlation under low and high *PROS1* expression conditions.

Gene Name	Low *PROS1* Expression Conditions			High *PROS1* Expression Conditions		
	Q	I^2 (%)	Q p-value	Q	I^2 (%)	Q p-value

AURKA	1.554544468	0	4.6×10^{-1}	2.19997303	9.1	0.332875572
AURKB	1.082992791	0	5.8×10^{-1}	0.184032671	0	0.91209025
PLK1	0.601032623	0	7.4×10^{-1}	0.408635478	0	0.815203308
PLK2	0.849965951	0	6.5×10^{-1}	0.475527465	0	0.78838894
PLK4	0.824557854	0	6.6×10^{-1}	0.585204674	0	0.746318865
CDK1	0.22156221	0	9.0×10^{-1}	0.951289347	0	0.621484273
CCNB1	0.950970563	0	6.2×10^{-1}	0.095838287	0	0.953210857
CCNB2	1.641436678	0	4.4×10^{-1}	0.342143024	0	0.842761304
CDC25C	0.742926641	0	6.9×10^{-1}	0.022791835	0	0.98866877
CDC20	1.505257497	0	4.7×10^{-1}	0.269778817	0	0.873812543
CDH1	1.690895071	0	4.3×10^{-1}	0.010583158	0	0.994722397
ANAPC2	1.280110624	0	5.3×10^{-1}	0.127234884	0	0.93836391
ANAPC7	0.412942655	0	8.1×10^{-1}	1.461250934	0	0.481607666
ANAPC10	0.262674894	0	8.8×10^{-1}	0.287474986	0	0.866115079
BUB1	0.657674093	0	7.2×10^{-1}	0.227647975	0	0.892415018
MAD2L1	0.670337686	0	7.2×10^{-1}	0.873469878	0	0.646142676
TTK	1.0818136	0	5.8×10^{-1}	0.035404144	0	0.982453689
NDC80	1.376233724	0	5.0×10^{-1}	2.617888328	23.6	0.270105093
NUF2	0.030430875	0	9.8×10^{-1}	0.675672998	0	0.713311905
SPC25	0.618432557	0	7.3×10^{-1}	0.171742908	0	0.917712192
CENPA	0.210812773	0	9.0×10^{-1}	1.695086946	0	0.428466179
CENPB	0.625816432	0	7.3×10^{-1}	2.080249108	3.8	0.35341066
CENPC	0.436549573	0	8.0×10^{-1}	1.469053444	0	0.479732452
CENPE	0.632124211	0	7.3×10^{-1}	0.256747611	0	0.879524547
CENPF	2.45245054	18.0	2.9×10^{-1}	0.537340963	0	0.764395096
MIS12	0.335535287	0	8.5×10^{-1}	0.566435291	0	0.753355805
ZWILCH	0.254590808	0	8.8×10^{-1}	0.053941929	0	0.973389504
ZWINT	1.117314618	0	5.7×10^{-1}	0.182543511	0	0.912769627
KNL1	1.095510177	0	5.8×10^{-1}	0.316086093	0	0.853813028
INCENP	2.1349831	6.3	3.4×10^{-1}	0.008597179	0	0.995710636

CDCA8	1.586014944	0	4.5×10^{-1}	0.106724789	0	0.948036387
BIRC5	0.347178726	0	8.4×10^{-1}	0.265177763	0	0.875825086
TPX2	1.332913904	0	5.1×10^{-1}	0.277504402	0	0.870443697
KIF11	0.660329155	0	7.2×10^{-1}	0.001341021	0	0.999329714
KIF18A	0.826759952	0	6.6×10^{-1}	0.003535009	0	0.998234057
KIF4A	0.800563628	0	6.7×10^{-1}	0.192769037	0	0.908114762
KIF23	1.460445668	0	4.8×10^{-1}	0.42249102	0	0.809575284
KIF15	1.074196043	0	5.8×10^{-1}	0.088791364	0	0.956575383
DYNC1H1	1.530890056	0	4.7×10^{-1}	0.173240663	0	0.917025195
DCTN1	1.418534235	0	4.9×10^{-1}	0.120916604	0	0.94133302
NUMA1	1.311305681	0	5.2×10^{-1}	0.147928789	0	0.92870476
TACC3	0.353746441	0	8.4×10^{-1}	0.227068069	0	0.892673814
CKAP5	1.829971079	0	4.0×10^{-1}	0.071734642	0	0.96476829
CLASP1	0.483419521	0	7.9×10^{-1}	0.003463282	0	0.998269858
MAPRE1	0.240408735	0	8.9×10^{-1}	0.027121062	0	0.986530999
CAMSAP3	0.340880502	0	8.4×10^{-1}	0.048944939	0	0.975824553
SMC1A	0.62711659	0	7.3×10^{-1}	0.088165249	0	0.956874893
SMC3	0.388214246	0	8.2×10^{-1}	1.238902655	0	0.538239674
RAD21	0.140001571	0	9.3×10^{-1}	2.095446583	4.5	0.350735363
STAG1	2.312837051	14.0	3.1×10^{-1}	0.000118875	0	0.999940564
ESCO1	1.841857806	0	4.0×10^{-1}	1.465858502	0	0.480499423
WAPL	1.43748191	0	4.9×10^{-1}	0.134631093	0	0.93490015
SMC2	0.001206857	0	1.0×10^{0}	0.089770091	0	0.956107384
SMC4	0.374787368	0	8.3×10^{-1}	0.027016107	0	0.986582771
NCAPD2	0.79017971	0	6.7×10^{-1}	0.629358062	0	0.730023152
NCAPG	0.398165948	0	8.2×10^{-1}	0.184479228	0	0.911886623
NCAPH	0.681599376	0	7.1×10^{-1}	0.034215519	0	0.983037747
NCAPD3	2.403039065	17.0	3.0×10^{-1}	2.482799198	19.4	0.288979479
RCC1	0.614122671	0	7.4×10^{-1}	0.006534137	0	0.996738263
GAS2L3	1.772095387	0	4.1×10^{-1}	7.68502× 10−6	0	0.999996157

AKT1	0.338638477	0	8.4×10^{-1}	0.073964165	0	0.963693402
HIF1A	1.520174535	0	4.7×10^{-1}	1.414991603	0	0.492876915
PFKFB4	0.020584178	0	9.9×10^{-1}	0.762068344	0	0.683154545
E2F1	0.160265923	0	9.2×10^{-1}	0.406630242	0	0.816021055
PTK2	0.157339976	0	9.2×10^{-1}	0.809986508	0	0.66698131
NRAS	0.559560143	0	7.6×10^{-1}	0.471519864	0	0.789970298
SNAI1	0.022419861	0	9.9×10^{-1}	2.196929257	8.9	0.333382557
TYRO3	0.025833824	0	9.9×10^{-1}	1.460259857	0	0.48184638
PDK1	0.725368705	0	7.0×10^{-1}	0.050922891	0	0.974859963
PFKFB3	0.610983921	0	7.4×10^{-1}	0.00735376	0	0.996329871
EPAS1	0.339607504	0	8.4×10^{-1}	0.186963623	0	0.910754583
PROM1	0.338050834	0	8.4×10^{-1}	1.206659631	0	0.546987234
KRAS	1.5502052	0	4.6×10^{-1}	0.581756253	0	0.747606786
IL6	0.005513938	0	1.0×10^{0}	0.139794737	0	0.932489518
MYC	1.270216473	0	5.3×10^{-1}	0.35219731	0	0.838535254
MKI67	0.948067143	0	6.2×10^{-1}	0.128067319	0	0.937973427
CD44	0.445532869	0	8.0×10^{-1}	1.289884583	0	0.524692821
VEGFA	2.385923489	16.0	3.0×10^{-1}	1.779580994	0	0.410741795
SRC	0.586255269	0	7.5×10^{-1}	1.562595856	0	0.457811419
SLC2A1	1.264207767	0	5.3×10^{-1}	0.064068903	0	0.968473216
HK2	0.909833394	0	6.3×10^{-1}	0.46991214	0	0.79060558
TWIST1	0.620791258	0	7.3×10^{-1}	0.057388801	0	0.971713374
CDH2	0.025928121	0	9.9×10^{-1}	0.070046806	0	0.965582818
TNF	0.113767721	0	9.4×10^{-1}	2.385168138	16.1	0.30343615
TGFB1	1.734301007	0	4.2×10^{-1}	2.127313269	5.9	0.345191261
CA9	0.52133888	0	7.7×10^{-1}	0.413632395	0	0.813169099
MMP9	0.521395451	0	7.7×10^{-1}	0.0434434	0	0.978512517
MMP2	0.013942145	0	9.9×10^{-1}	1.835508067	0	0.399415107
SNAI2	0.134803456	0	9.3×10^{-1}	0.068466718	0	0.966345973

Supplementary Table S7. Heterogeneity statistics, including Cochran's Q-value, Cochran's Q p-value, and I^2 value as a percentage, for all genes in ovarian serous cystadenocarcinoma human RNA-sequencing data that showed a statistically significant correlation under low and high *PROS1* expression conditions.

Gene Name	Low *PROS1* Expression Conditions			High *PROS1* Expression Conditions		
	Q	I^2 (%)	Q p-value	Q	I^2 (%)	Q p-value
AURKA	0.184409304	0	0.911918504	0.280737334	0	0.869037791
AURKC	0.495047264	0	0.78073177	0.412361685	0	0.813685914
PLK1	0.00045065	0	0.9997747	0.351002064	0	0.839036531
PLK2	0.001227008	0	0.999386684	0.029724122	0	0.985247834
CDK1	0.179395119	0	0.914207637	0.078686113	0	0.961420831
CCNB1	0.000677905	0	0.999661105	0.040702699	0	0.979854342
CDC20	0.195080496	0	0.907065833	0.050354478	0	0.975137064
ANAPC1	0.404877594	0	0.816736468	0.103153007	0	0.949730989
ANAPC2	6.07014× 10−7	0	0.999999696	1.960742708	0	0.375171751
ANAPC4	0.017798699	0	0.991140133	0.163257161	0	0.921614201
ANAPC5	0.016874964	0	0.991598014	0.000611166	0	0.999694464
ANAPC10	2.523308901	20.7	0.283185123	0.096418635	0	0.9529343
BUB1B	1.190292309	0	0.551481958	6.43679× 10−7	0	0.999999678
BUB3	0.246338068	0	0.884114205	1.299175209	0	0.52226111
MAD2L1BP	0.661759841	0	0.718291416	1.214634429	0	0.54481052
NDC80	0.082002077	0	0.959828133	0.22147691	0	0.895172846
NUF2	0.293224147	0	0.863628937	0.539294592	0	0.763648789
SPC24	0.027328058	0	0.9864289	0.227405219	0	0.892523344
CENPB	0.111392516	0	0.94582638	0.187499527	0	0.910510577
CENPE	2.74607× 10−6	0	0.999998627	0.434447012	0	0.804750083
CENPF	0.073010584	0	0.964152992	0.51443185	0	0.773201242
MIS12	0.00529751	0	0.99735475	0.508025564	0	0.775681887
ZWILCH	0.330069886	0	0.847864077	0.030355536	0	0.984936834
ZWINT	0.28962693	0	0.865183665	2.584142548	22.6	0.274701212

KNL1	0.296662883	0	0.862145317	0.00164753	0	0.999176574
INCENP	0.143273944	0	0.930868766	0.049794559	0	0.975410102
CDCA8	0.379966206	0	0.826973107	0.223482631	0	0.894275563
KIF11	0.008695336	0	0.99566177	0.361517205	0	0.834636813
KIF2C	0.051224959	0	0.974712738	0.164876623	0	0.920868243
KIF4A	0.296588173	0	0.862177523	0.009748851	0	0.995137435
KIF23	0.051806812	0	0.974429209	0.233817089	0	0.889666554
KIF15	0.009668737	0	0.995177298	0.000447851	0	0.9997761
KIFC1	0.837306973	0	0.657932138	0.587356752	0	0.745516229
TACC3	0.309774363	0	0.856511802	0.210186876	0	0.900240402
CKAP5	0.091243738	0	0.955403161	0.61110258	0	0.736717118
CAMSAP3	0.00220788	0	0.998896669	0.007128945	0	0.996441873
WAPL	0.108335058	0	0.947273397	0.169895207	0	0.918560413
NCAPD2	0.755631995	0	0.685356596	0.081089666	0	0.960266112
NCAPH	0.097286595	0	0.952520836	0.398907925	0	0.819177933
NCAPD3	0.088885297	0	0.956530457	0.396011048	0	0.820365322
GAS2L3	2.216838086	9.7	0.330080392	0.657966886	0	0.719654932
CD8A	1.032770205	0	0.596673568	0.001741321	0	0.999129718
HIF3A	0.08154472	0	0.960047651	0.20878673	0	0.900870857
IL1B	1.976183465	0	0.372286436	0.25331357	0	0.881036005
PFKFB4	0.102431171	0	0.950073826	0.443955501	0	0.800933185
CASP3	0.040681544	0	0.979864706	0.971131701	0	0.615348903
NRAS	0.307173905	0	0.857626188	0.569232215	0	0.752303003
SNAI1	0.073134584	0	0.964093216	0.349358177	0	0.839726456
MERTK	0.018152359	0	0.990964885	4.49162×10^{-5}	0	0.999977542
STAT3	0.087992188	0	0.956957695	0.100248991	0	0.951111008
VIM	0.369172656	0	0.83144816	1.278126294	0	0.527786651
MKI67	0.659271956	0	0.719185485	0.00609618	0	0.996956551
SRC	0.463909679	0	0.792981934	1.670953781	0	0.43366762
SLC2A1	1.385845481	0	0.500112233	0.841189092	0	0.656656291

NOTCH1	0.426283961	0	0.808041403	1.4943769	0	0.473696504
HK2	2.355106388	15.0	0.308031511	0.027949274	0	0.986122555
TWIST1	0.929187186	0	0.628390435	0.471206358	0	0.790094138
CDH2	0.311658965	0	0.855705091	1.369866773	0	0.50412381
TNF	2.210581531	9.5	0.331114592	0.017725424	0	0.991176446
CA9	0.406632241	0	0.81602024	0.275333262	0	0.871389138
MMP9	0.030941186	0	0.984648462	1.791773791	0	0.408245367
MMP7	0.044625612	0	0.977934283	0.520180982	0	0.770981816
SNAI2	0.019110411	0	0.9904903	5.64234× 10−5	0	0.999971789

Supplementary Table S8. Heterogeneity statistics, including Cochran's Q-value, Cochran's Q p-value, and I^2 value as a percentage, for all genes in pancreatic ductal adenocarcinoma human RNA-sequencing data that showed a statistically significant correlation under low and high *PROS1* expression conditions.

**Gene Name**	**Low *PROS1* Expression Conditions**			**High *PROS1* Expression Conditions**		
	**Q**	**I^2 (%)**	**Q p-value**	**Q**	**I^2 (%)**	**Q p-value**
ANAPC10	0.7418	0	6.9× 10−1	0.422257001	0	0.80967
ANAPC2	2.6038	2.3×10^1	2.7× 10−1	0.073657787	0	0.963841
ANAPC4	1.4542	0	4.8× 10−1	0.628955623	0	0.7301701
AURKC	0.1940	0	9.1× 10−1	0.810894085	0	0.6666787
CAMSAP3	0.080228655	0	0.960679601	0.499294132	0	0.7790757
CENPA	1.2650	0	5.3× 10−1	0.215636298	0	0.8977908
CLASP1	0.380105419	0	0.826915546	0.003609912	0	0.9981967
CLASP2	1.101899457	0	0.576402125	0.000262191	0	0.9998689
ESCO1	1.538129913	0	0.463446208	0.33312584	0	0.8465695
GAS2L3	1.589315646	0	0.451735782	1.584777606	0	0.4527619
KNL1	2.544918077	21.41200859	0.280141894	2.352917206	14.99913404	0.3083689
MAPRE1	0.19109226	0	0.908876434	1.494417487	0	0.4736869
NCAPD2	0.925356287	0	0.629595239	0.449750839	0	0.7986157
PDS5B	1.831745058	0	0.400167316	0.782669908	0	0.6761536

SMC1A	0.169421201	0	0.91877814	0.133292577	0	0.935526
TOP2B	1.665339007	0	0.434886803	0.242388053	0	0.8858621
WAPL	0.048650786	0	0.975968085	1.188950766	0	0.551852
ZWILCH	2.035351296	1.736864596	0.361434065	0.584905884	0	0.7464304

Supplementary Table S9. Heterogeneity statistics, including Cochran's Q-value, Cochran's Q p-value, and I^2 value as a percentage, for all genes in NSCLC lung adenocarcinoma human RNA-sequencing data that were eliminated due to high heterogeneity between cohorts under low and high *PROS1* expression conditions.

Gene Name	Low *PROS1* Expression Conditions			High *PROS1* Expression Conditions		
	Q	I^2 (%)	Q P-value	Q	I^2 (%)	Q P-value
AURKC	11.27680849	82.26448555	0.003558542	1.53185913	0	0.464901574
ANAPC1	16.00268777	87.50209946	0.000335012	1.084452627	0	0.581452315
ANAPC4	14.95239033	86.62421221	0.000566408	0.832433851	0	0.659537184
ANAPC5	0.417652422	0	0.811536259	11.95737348	83.27391878	0.002532149
BUB1B	2.997660962	33.28131416	0.223391268	0.027583174	0	0.986303081
MAD1L1	32.33386172	93.81453407	9.52338× 10−8	1.529157227	0	0.465530058
SPC24	1.861678378	0	0.394222744	3.113754641	35.76886331	0.210793284
KIF2C	2.894895154	30.91286926	0.235169775	0.000126256	0	0.999936874
KIFC1	2.792097959	28.36927538	0.247573201	0.104679051	0	0.9490066
CLASP2	3.118839702	35.87358791	0.210258017	0.530000925	0	0.767205595
STAG2	5.17792188	61.37446554	0.075098031	0.041576081	0	0.979426542
ESCO2	2.913541806	31.35502652	0.2329874	5.139339766	61.08449546	0.076560815
PDS5A	13.6358377	85.33276764	0.001093995	1.236876012	0	0.53878536
PDS5B	15.00351452	86.66978996	0.000552113	0.240083768	0	0.88688329
TOP2A	2.678679385	25.33634256	0.262018624	0.730067626	0	0.694173178
TOP2B	4.679021608	57.25602129	0.096374773	2.956856837	32.36060756	0.227995721
NCAPH2	14.16796618	85.88364784	0.000838427	1.568313945	0	0.456504385
CASP9	16.97376543	88.21711065	0.000206155	3.800879433	47.38059875	0.149502866
CD8A	6.585007652	69.62797758	0.037160689	9.068815406	77.94640303	0.010733263

MAPK1	5.189953913	61.46401233	0.074647596	6.687975342	70.09558352	0.035295928
HIF3A	2.742180645	27.06534475	0.253830052	1.081836992	0	0.582213246
IL1B	1.172357777	0	0.556449483	3.757961353	46.77965492	0.152745724
CASP3	18.45684183	89.16390996	9.82082× 10−5	11.07334466	81.93860968	0.003939615
PIK3CA	3.094335775	35.36577329	0.212849936	0.939879559	0	0.625039907
HRAS	0.0695442	0	0.965825503	4.112646037	51.36950805	0.127923478
MERTK	3.809360685	47.49775184	0.148870223	8.663351417	76.91424596	0.013145501
STAT3	2.669171489	25.07038201	0.263267213	3.719835697	46.23418444	0.15568542
CD4	2.925248113	31.62973113	0.231627672	6.722018445	70.24703196	0.034700221
PECAM1	4.307580807	53.57022678	0.116043472	12.25257565	83.67690143	0.002184676
VIM	3.888576984	48.56730345	0.143088996	0.434734931	0	0.80463424
CDK2	3.366271415	40.58708425	0.185790477	0.056991078	0	0.97190663
AXL	4.513454918	55.68804749	0.104692535	1.321625246	0	0.5164315
NOTCH1	3.729779107	46.37752149	0.154913319	0.658255219	0	0.719551189
MMP7	1.454821137	0	0.483158478	25.29224306	92.09243721	3.22002× 10−6

Supplementary Table S10. Heterogeneity statistics, including Cochran's Q-value, Cochran's Q p-value, and I^2 value as a percentage, for all genes in ovarian serous cystadenocarcinoma human RNA-sequencing data that were eliminated due to high heterogeneity between cohorts under low and high *PROS1* expression conditions.

**Gene Name**	**Low *PROS1* Expression Conditions**			**High *PROS1* Expression Conditions**		
	**Q**	**I^2 (%)**	**Q P-value**	**Q**	**I^2 (%)**	**Q P-value**
AURKB	8.85307183	77.408971	0.0119558	0.286882099	0	0.8663719
PLK4	0.133154224	0	0.9355908	8.489395583	76.441197	0.0143401
CCNB2	11.05230155	81.904222	0.0039813	0.555244471	0	0.757583
CDC25C	0.081771097	0	0.959939	9.340305987	78.587425	0.0093708
CDH1	11.0318829	81.870729	0.0040221	0.070985127	0	0.9651299
ANAPC7	8.793999982	77.257221	0.0123142	8.18672858	75.570217	0.016683
BUB1	0.510048213	0	0.7748978	10.40263363	80.774099	0.0055093

MAD1L1	0.677995676	0	0.712484	9.527985264	79.009203	0.0085315
MAD2L1	0.194234212	0	0.9074497	12.86484322	84.453755	0.0016086
TTK	0.29808327	0	0.8615332	9.072810078	77.956113	0.0107118
SPC25	0.11894169	0	0.942263	24.50883089	91.839676	4.764×10^{-6}
CENPA	0.549582848	0	0.7597306	10.77104764	81.431704	0.0045824
CENPC	0.597990951	0	0.7415628	10.20221057	80.396405	0.00609
BIRC5	0.882412455	0	0.64326	30.76148163	93.498363	2.09×10^{-7}
TPX2	1.573485223	0	0.4553256	11.17193299	82.097995	0.0037501
KIF18A	12.49937646	83.999202	0.0019311	0.056085996	0	0.9723466
DYNC1H1	0.848323885	0	0.6543179	12.19292399	83.597044	0.0022508
DCTN1	12.32584663	83.773934	0.0021061	8.570549463	76.664273	0.0137698
NUMA1	2.003859784	0.1926174	0.3671702	20.35480353	90.174309	3.802×10^{-5}
CLASP1	13.46110136	85.142375	0.0011939	0.219988664	0	0.8958392
CLASP2	12.52080158	84.026582	0.0019105	0.000178647	0	0.9999107
MAPRE1	0.012456399	0	0.9937912	15.06754994	86.726442	0.0005347
SMC1A	14.64985815	86.347991	0.0006589	10.25370242	80.49485	0.0059352
SMC3	0.597401861	0	0.7417812	11.84574312	83.116298	0.0026775
RAD21	2.575525797	22.345954	0.2758873	10.59486774	81.122936	0.0050044
STAG1	19.31767887	89.646789	6.386×10^{-5}	0.089312176	0	0.9563263
STAG2	8.182049716	75.556247	0.0167221	11.8030174	83.05518	0.0027353
ESCO1	10.00169979	80.003399	0.0067322	11.34205669	82.366514	0.0034443
ESCO2	12.43975394	83.922512	0.0019895	0.250045441	0	0.8824769
PDS5A	8.517596388	76.519197	0.0141393	8.889642163	77.501907	0.0117392
PDS5B	10.78734456	81.459756	0.0045453	0.878080664	0	0.6446548
TOP2A	9.642075526	79.257578	0.0080584	0.591007367	0	0.7441567
TOP2B	8.389663773	76.161142	0.0150733	0.040236036	0	0.980083
SMC2	9.430810842	78.792916	0.0089562	9.385064871	78.689545	0.0091635
SMC4	0.096653366	0	0.9528225	8.213718585	75.650493	0.0164594
NCAPG	0.051674959	0	0.9744935	8.816488516	77.315232	0.0121765
NCAPH2	10.3538006	80.683422	0.0056455	0.389700597	0	0.8229578

RCC1	9.626036739	79.223017	0.0081233	10.98580568	81.79469	0.0041159
CASP9	9.222261384	78.313345	0.0099406	2.106008653	5.0336286	0.348888
AKT1	0.158033586	0	0.9240244	15.65522452	87.224712	0.0003986
MAPK1	0.067028266	0	0.9670412	8.788942029	77.244133	0.0123454
HIF1A	0.000530091	0	0.999735	11.41067176	82.472548	0.0033282
E2F1	0.243044424	0	0.8855714	15.28018553	86.911154	0.0004808
PIK3CA	0.003100522	0	0.9984509	20.46094486	90.22528	3.605× 10−5
PTK2	8.321925954	75.967102	0.0155925	2.488245326	19.622073	0.2881936
HRAS	10.24879435	80.485509	0.0059498	10.04338809	80.086401	0.0065933
TYRO3	3.222885536	37.943809	0.1995994	10.56768058	81.074371	0.0050729
PDK1	10.48904558	80.932488	0.0052763	0.352574458	0	0.8383771
PFKFB3	10.28153051	80.547643	0.0058532	8.035480097	75.110386	0.0179936
CD4	8.689227359	76.982994	0.0129765	0.372941365	0	0.8298829
EPAS1	0.00441762	0	0.9977936	18.95085316	89.446385	7.671× 10−5
PECAM1	0.386087091	0	0.8244461	8.758697499	77.165555	0.0125335
PROM1	8.740553508	77.118154	0.0126477	5.695674932	64.885636	0.0579695
KRAS	0.547283312	0	0.7606046	3.855595514	48.127339	0.1454682
IL6	0.397126449	0	0.8199079	9.859317827	79.714621	0.007229
MYC	0.407731647	0	0.8155718	14.28820204	86.002438	0.0007895
CD44	8.428955381	76.272267	0.01478	10.88169451	81.62051	0.0043358
CDK2	0.024739408	0	0.9877065	8.624061854	76.809072	0.0134063
VEGFA	0.375392226	0	0.8288666	3.592897815	44.334626	0.1658869
AXL	0.466206863	0	0.7920716	16.13476015	87.604402	0.0003136
TGFB1	0.115635481	0	0.943822	10.63050419	81.186217	0.004916
MMP2	0.017532742	0	0.9912719	16.04431714	87.534527	0.0003281

Supplementary Table S11. Heterogeneity statistics, including Cochran's Q-value, Cochran's Q p-value, and I^2 value as a percentage, for all genes in pancreatic ductal adenocarcinoma human RNA-sequencing data that were eliminated due to high heterogeneity between cohorts under low and high *PROS1* expression conditions.

**Gene Name**	**Low *PROS1***	**High *PROS1***

	Expression Conditions			Expression Conditions		
	Q	I^2 (%)	Q p-value	Q	I^2 (%)	Q p-value
AURKA	28.82062651	93.060526	5.517×10^{-7}	1.851035434	0	0.3963262
AURKB	5.855074735	65.841597	0.0535287	1.163732316	0	0.5588545
PLK1	8.589849245	76.716704	0.0136376	0.514676077	0	0.7731068
PLK2	0.021150484	0	0.9894805	7.479349319	73.259706	0.0237618
PLK4	36.33667812	94.49592	1.287×10^{-8}	0.519384278	0	0.771289
CCNB1	36.27198718	94.486103	1.329×10^{-8}	1.424419113	0	0.4905591
CCNB2	15.13481398	86.785434	0.000517	1.172503131	0	0.556409
CDC25C	4.956411434	59.648225	0.0838936	0.132436122	0	0.9359268
CDC20	6.26703312	68.086973	0.0435643	1.003259792	0	0.6055429
ANAPC1	41.61477989	95.194015	9.193×10^{-10}	25.44767126	92.140735	2.979×10^{-6}
ANAPC5	27.09913426	92.61969	1.305×10^{-6}	19.82411168	89.911276	4.957×10^{-5}
ANAPC7	26.11746388	92.342289	2.131×10^{-6}	11.69577692	82.899811	0.002886
BUB1	16.78689014	88.085941	0.0002263	4.486752481	55.42433	0.1060997
BUB1B	18.0878233	88.942838	0.0001181	0.343506589	0	0.8421869
BUB3	14.56964729	86.272832	0.0006859	0.389933801	0	0.8228619
MAD1L1	8.48342273	76.42461	0.014383	0.008684356	0	0.9956672
MAD2L1	25.46167784	92.145058	2.958×10^{-6}	0.332224859	0	0.846951
MAD2L1BP	20.1243718	90.061802	4.266×10^{-5}	16.7106484	88.031584	0.0002351
TTK	33.81037002	94.084655	4.552×10^{-8}	0.400544162	0	0.818508
NDC80	42.95588197	95.34406	4.702×10^{-10}	0.600739567	0	0.7405443
NUF2	24.44996497	91.820029	4.906×10^{-6}	0.598533868	0	0.7413615
SPC24	3.204928459	37.596111	0.2013996	3.163893942	36.786756	0.2055745
SPC25	28.65249601	93.019805	6.001×10^{-7}	15.86899835	87.39681	0.0003582
CENPB	22.41990217	91.079354	1.354×10^{-5}	0.086246342	0	0.9577934
CENPC	4.109719564	51.334879	0.1281108	1.024059754	0	0.5992779
CENPE	29.40586326	93.198635	4.117×10^{-7}	0.573842424	0	0.7505709
CENPF	20.00771921	90.003858	4.523×10^{-5}	0.255960547	0	0.8798707
MIS12	3.029775718	33.988513	0.2198328	0.845954997	0	0.6550934

ZWINT	16.07954808	87.561839	0.0003224	0.927279501	0	0.6289901
INCENP	25.09687625	92.030881	$3.55\times 10{-}6$	1.309736234	0	0.5195106
CDCA8	6.087491061	67.145742	0.0476561	0.573109648	0	0.7508459
BIRC5	10.77178994	81.432984	0.0045807	0.966992232	0	0.6166238
TPX2	32.10260413	93.769976	$1.069\times 10{-}7$	0.684448611	0	0.7101889
KIF11	23.08770095	91.337379	$9.695\times 10{-}6$	0.704762855	0	0.7030119
KIF2C	27.17611972	92.640598	$1.255\times 10{-}6$	0.260461949	0	0.8778926
KIF18A	17.91964186	88.839063	0.0001285	0.054384265	0	0.9731742
KIF4A	9.849146138	79.693671	0.0072658	0.971480025	0	0.6152417
KIF23	11.49426231	82.600014	0.0031919	1.03431955	0	0.5962115
KIF15	13.95961366	85.672956	0.0009305	0.149399963	0	0.9280219
KIFC1	25.03153755	92.010079	$3.668\times 10{-}6$	0.445168406	0	0.8004476
DYNC1H1	28.37922157	92.95259	$6.879\times 10{-}7$	0.543859904	0	0.7619076
DCTN1	9.239159927	78.35301	0.0098569	14.14051013	85.856239	0.00085
NUMA1	20.56087502	90.272788	$3.43\times 10{-}5$	0.773268979	0	0.6793394
TACC3	4.381085622	54.349214	0.111856	50.30795079	96.024485	$1.191\times 10{-}11$
CKAP5	22.98331802	91.298036	$1.021\times 10{-}5$	13.13351955	84.77179	0.0014063
SMC3	1.418334456	0	0.4920538	3.114323149	35.780588	0.2107334
RAD21	19.26731166	89.619725	$6.549\times 10{-}5$	0.740619505	0	0.6905204
STAG1	4.193358954	52.305538	0.1228637	1.209021276	0	0.5463417
STAG2	25.58031417	92.181488	$2.788\times 10{-}6$	0.839205186	0	0.657308
ESCO2	26.03147851	92.316994	$2.225\times 10{-}6$	0.1217614	0	0.9409355
PDS5A	22.78916081	91.223898	$1.126\times 10{-}5$	0.752304377	0	0.6864978
TOP2A	8.587176308	76.709457	0.0136558	1.763235024	0	0.4141125
SMC2	24.53321048	91.847785	$4.706\times 10{-}6$	2.463018878	18.798836	0.2918517
SMC4	50.39089888	96.031029	$1.142\times 10{-}11$	15.50100995	87.097615	0.0004305
NCAPG	12.89902581	84.494953	0.0015813	1.02714985	0	0.5983527
NCAPH	8.805375027	77.286601	0.0122444	1.190534063	0	0.5514153
NCAPD3	42.07272796	95.246327	$7.312\times 10{-}10$	1.992142446	0	0.3693276
NCAPH2	14.1860613	85.901654	0.0008309	0.093234224	0	0.9544528

RCC1	20.47610156	90.232516	3.578× 10−5	0.042887016	0	0.9787848

Supplementary Table S12. The list of 49 genes associated with oncogenic signaling and 78 genes associated with cell cycle signaling evaluated for NSCLC lung adenocarcinoma and ovarian serous cystadenocarcinoma. Genes evaluated for pancreatic ductal adenocarcinoma are highlighted in light gray, since the evaluation of the remaining genes has been published.

AURKA	*BUB1B*	*CDCA8*	*KIF15*	*CASP9*	*PDK1*	*NOTCH1*
AURKB	*BUB3*	*BIRC5*	*KIFC1*	*AKT1*	*STAT3*	*HK2*
AURKC	*MAD1L1*	*TPX2*	*DYNC1H1*	*CD8A*	*PFKFB3*	*TWIST1*
PLK1	*MAD2L1*	*KIF11*	*DCTN1*	*MAPK1*	*CD4*	*CDH2*
PLK2	*MAD2L1BP*	*KIF2C*	*NUMA1*	*HIF3A*	*EPAS1*	*TNF*
PLK4	*TTK*	*KIF18A*	*TACC3*	*IL1B*	*PECAM1*	*TGFB1*
CDK1	*NDC80*	*KIF4A*	*CKAP5*	*HIF1A*	*PROM1*	*CA9*
CCNB1	*NUF2*	*KIF23*	*CLASP1*	*PFKFB4*	*KRAS*	*MMP9*
CCNB2	*SPC24*	*SMC2*	*CLASP2*	*CASP3*	*IL6*	*MMP7*
CDC25C	*SPC25*	*SMC4*	*MAPRE1*	*E2F1*	*VIM*	*MMP2*
CDC20	*CENPA*	*NCAPD2*	*CAMSAP3*	*PIK3CA*	*MYC*	*SNAI2*
CDH1 (FZR1)	*CENPB*	*NCAPG*	*SMC1A*	*PTK2*	*MKI67*	*F9*
ANAPC1	*CENPC*	*NCAPH*	*SMC3*	*NRAS*	*CD44*	*SNAI1*
ANAPC2	*CENPE*	*NCAPD3*	*RAD21*	*SLC2A1*	*CDK2*	*HRAS*
ANAPC4	*CENPF*	*NCAPH2*	*STAG1*	*PDS5A*	*VEGFA*	*MERTK*
ANAPC5	*MIS12*	*ESCO2*	*STAG2*	*PDS5B*	*SRC*	*TYRO3*
ANAPC7	*ZWILCH*	*RCC1*	*ESCO1*	*TOP2A*	*AXL*	
ANAPC10	*ZWINT*	*GAS2L3*	*TOP2B*			
WAPL	*BUB1*	*INCENP*	*KNL1*			
